# Targeting the Active Rhizosphere Microbiome of *Trifolium pratense* in Grassland Evidences a Stronger-Than-Expected Belowground Biodiversity-Ecosystem Functioning Link

**DOI:** 10.3389/fmicb.2021.629169

**Published:** 2021-02-01

**Authors:** Sara Fareed Mohamed Wahdan, Anna Heintz-Buschart, Chakriya Sansupa, Benjawan Tanunchai, Yu-Ting Wu, Martin Schädler, Matthias Noll, Witoon Purahong, François Buscot

**Affiliations:** ^1^Department of Soil Ecology, Helmholtz Centre for Environmental Research-UFZ, Halle (Saale), Germany; ^2^Department of Biology, Leipzig University, Leipzig, Germany; ^3^Department of Botany, Faculty of Science, Suez Canal University, Ismailia, Egypt; ^4^German Centre for Integrative Biodiversity Research (iDiv) Halle-Jena-Leipzig, Leipzig, Germany; ^5^Department of Forestry, National Pingtung University of Science and Technology, Pingtung, Taiwan; ^6^Department of Community Ecology, Helmholtz Centre for Environmental Research-UFZ, Halle (Saale), Germany; ^7^Institute for Bioanalysis, Coburg University of Applied Sciences and Arts, Coburg, Germany

**Keywords:** active microbiome, rhizosphere, biodiversity-ecosystem functioning, GCEF, BrdU

## Abstract

The relationship between biodiversity and ecosystem functioning (BEF) is a central issue in soil and microbial ecology. To date, most belowground BEF studies focus on the diversity of microbes analyzed by barcoding on total DNA, which targets both active and inactive microbes. This approach creates a bias as it mixes the part of the microbiome currently steering processes that provide actual ecosystem functions with the part not directly involved. Using experimental extensive grasslands under current and future climate, we used the bromodeoxyuridine (BrdU) immunocapture technique combined with pair-end Illumina sequencing to characterize both total and active microbiomes (including both bacteria and fungi) in the rhizosphere of *Trifolium pratense*. Rhizosphere function was assessed by measuring the activity of three microbial extracellular enzymes (β-glucosidase, N-acetyl-glucosaminidase, and acid phosphatase), which play central roles in the C, N, and P acquisition. We showed that the richness of overall and specific functional groups of active microbes in rhizosphere soil significantly correlated with the measured enzyme activities, while total microbial richness did not. Active microbes of the rhizosphere represented 42.8 and 32.1% of the total bacterial and fungal taxa, respectively, and were taxonomically and functionally diverse. Nitrogen fixing bacteria were highly active in this system with 71% of the total operational taxonomic units (OTUs) assigned to this group detected as active. We found the total and active microbiomes to display different responses to variations in soil physicochemical factors in the grassland, but with some degree of resistance to a manipulation mimicking future climate. Our findings provide critical insights into the role of active microbes in defining soil ecosystem functions in a grassland ecosystem. We demonstrate that the relationship between biodiversity-ecosystem functioning in soil may be stronger than previously thought.

## Introduction

Microbial communities in soil exhibit high phylogenetic (Tringe et al., 2005; Hug et al., 2016), taxonomic (Fierer et al., 2007), and functional (Prosser, 2002; Escalas et al., 2019) diversity and contribute to several ecosystem services (Saccá et al., 2017) by maintaining multiple functions (Jurburg and Salles, 2015; Delgado-Baquerizo et al., 2016; Soliveres et al., 2016). Most soil functions are more likely to be driven by the active microbial diversity at each specific time point (Bastida et al., 2016). However, in soils, large proportions of the microorganisms are not active at each time point (Blagodatskaya and Kuzyakov, 2013). Therefore, linking diversity of microbes to their contribution to soil functions during biodiversity-ecosystem functioning (BEF) studies remains controversial, as it requires distinguishing metabolically active microbes within the belowground ecosystem compartment (Carini et al., 2016).

The advent of high-throughput sequencing technology resulted in revealing the taxonomic diversity and composition of soil microbial communities. Most of these approaches detect the total genomic DNA persisting in soils, which includes a mixture of (i) relic or “extracellular” DNA able to persist many years in soils (Nielsen et al., 2007), (ii) DNA in non-intact cells, (iii) DNA of potentially active microbes that may trigger into activity within minutes to few hours by trace quantities of specific nutrients (De Nobili et al., 2001; Blagodatskaya and Kuzyakov, 2013), (iv) DNA of dormant microbes, and (v) DNA of living cells—intact, capable of reproduction, and metabolically active (Emerson et al., 2017). Not differentiating between these fractions leads to biased estimates of really active soil microbial diversity (Carini et al., 2016). To avoid such biases and only capture the active microbial communities, many approaches have been proposed, including RNA sequencing (Bowsher et al., 2019), metaproteomics (Williams et al., 2010), active cell staining (Bowsher et al., 2019), stable isotope probing (Dumont and Murrell, 2005), quantitative multi-isotope imaging mass spectrometry (MIMS) (Lechene et al., 2006), bio-orthogonal non-canonical amino acid tagging (BONCAT) (Hatzenpichler et al., 2016), and the viability indicator propidium monoazide (PMA) (Carini et al., 2016). An alternative approach for distinguishing active microbes within an ecosystem is the incorporation of the thymidine analog, bromodeoxyuridine (BrdU), into replicating cells during DNA synthesis, labeling actively growing microorganisms, and isolating the BrdU-labeled DNA by BrdU immunocapture using specific anti-BrdU antibodies (Borneman, 1999; Urbach et al., 1999). BrdU incorporation has been shown to successfully detect active bacteria in microcosms (Bravo et al., 2013; Kelly et al., 2016) and in natural habitats such as freshwater lakes (Grubisic et al., 2017), marine water (Galand et al., 2013; Taniguchi et al., 2015), sewage (Walters and Field, 2006), crop lands (Hjort et al., 2007), forest soil (Goldfarb et al., 2011), arctic soil (Mcmahon et al., 2011), fallow soil, and bacterial association with arbuscular mycorrhizal hyphae (Artursson and Jansson, 2003) as well as active fungi in forest soils (Allison et al., 2007; Allison and Treseder, 2008) and leaf litters (Allison et al., 2007). One potential disadvantage of BrdU-immunocapture technique is that not all microbial taxa are able to take up and incorporate BrdU (Mcmahon et al., 2011); however, a previous study (Hellman et al., 2011) showed that 18 out of 23 studied bacterial strains are able to incorporate BrdU, and generally, all bacterial phyla can be detected. No information regarding BrdU uptake capacity by fungal taxa is available.

Comparisons of active and total microbial communities have been done in many ecosystems including terrestrial and aquatic habitats (Baldrian et al., 2012; Romanowicz et al., 2016; Cardoso et al., 2017; Nawaz et al., 2018; Li et al., 2019). They revealed significant differences between these two communities, which are shaped partly by similar (Romanowicz et al., 2016; Nawaz et al., 2019) but also different (Rajala et al., 2011; Baldrian et al., 2012; Zhang et al., 2014) environmental factors. Soil physicochemical factors, climate change factors such as warming and altered precipitation patterns are known to modify ecosystem properties and processes and may affect the rhizosphere microbial diversity and community composition (Wieland et al., 2001; Garbeva et al., 2007; Drigo et al., 2008; Philippot et al., 2013; Alkorta et al., 2017). However, we still do not fully understand how the total and the active microbial communities in rhizosphere respond to such changes.

Soil functions can be assessed by various indicators. One of those biological indicators is extracellular enzyme activity (EEA) (Sinsabaugh et al., 2008; Steinauer et al., 2015; Bastida et al., 2016; Creamer et al., 2016). Production of microbial extracellular enzymes is higher in the biologically active rhizosphere zone as compared to the bulk soil (Joshi et al., 2018). Microbial extracellular enzymes are considered the proximate agents of organic matter break down and mineralization. Also, they have a protective function through oxidizing toxic substances (Gianfreda, 2015; Joshi et al., 2018). Consequently, EEA contributes to the supporting and regulating ecosystem services carried out by active microbes (Bodelier, 2011; Joshi et al., 2018). Although previous studies proved that the active diversity more accurately reflects ecosystem functionality than total diversity (Bastida et al., 2016; De Vrieze et al., 2018), to our knowledge, no studies to date have compared the links between total and active microbial diversity and soil ecosystem functions in the rhizosphere soil in grasslands.

In the present study, we used BrdU immunocapture combined with Illumina rRNA operon amplicon sequencing to characterize the total and active rhizosphere soil microbiome of *Trifolium pratense* (red clover) in a grassland ecosystem. *Trifolium pratense* is one of the most important forage legumes in the world and is adapted to many edaphic and climatic conditions (Taylor and Smith, 1979). It maintains high pasture quality under low fertilization by providing significant nitrogen input via symbiotic nitrogen fixation (Thilakarathna et al., 2018). To investigate the edaphic and climatic factors that shape the total and active rhizosphere microbiomes, we conducted the experiment at the Global Change Experimental Facility (GCEF) under both ambient conditions and a future climate scenario expected in 50–70 years from now in Central Germany (Schädler et al., 2019). In addition, we measured the activity of three microbial extracellular enzymes (β-glucosidase, N-acetyl-glucosaminidase, and acid phosphatase) in the same rhizosphere soil to be considered as indicators of ecosystem functions displayed by rhizosphere microbial communities for C, N, and P acquisition in this system. To get insights into the relationship between biodiversity–ecosystem functioning, we linked the measured EEA with two indices of microbial biodiversity: total microbial richness and active microbial richness and with the compositions of active and total communities. Our specific goals were to (i) estimate the proportion of active microbes relative to the total rhizosphere microbiome, (ii) study the responses of total and active microbiome to a manipulated future climate, and (iii) identify the possible links between total and active microbiomes and the soil ecosystem function. We expected differences in richness and community composition as well as the environmental drivers of total and active rhizosphere soil microbiomes. We hypothesized that the relationship between biodiversity–ecosystem functioning obtained from active rhizosphere soil microbiome to be stronger than the one from the total rhizosphere microbiome.

## Materials and Methods

### Study Site, Experimental Design, and Sampling Time

The study was conducted during summer (July) 2018 on the Global Change Experimental Facility (GCEF) that is settled in the field research station of the Helmholtz Centre for Environmental Research in Bad Lauchstädt, Saxony-Anhalt, Germany (51°22’60 N, 11°50’60 E, 118 m a.s.l.). The area is characterized by a sub-continental climate and prevailing West winds. Mean annual precipitation averages at 489 mm (1896–2013) and 525 mm (1993–2013) and mean temperature at 8.9°C (1896–2013), 9.7°C (1993–2013). The soil of the study site is a Haplic Chernozem characterized by a high content of organic carbon down to a depth of more than 40 cm and a high water holding capacity (Altermann et al., 2005). The GCEF consists of 50 plots with a size of 16 × 24 m each (Supplementary Figure S1). These 50 plots were randomly assigned to one of five land-use treatments (conventional farming, organic farming, intensively managed grassland, extensively managed grassland used meadow, and extensively managed grassland used pasture). Half of the plots (25 plots) are subjected to the ambient and future climate scenarios, respectively. The future climate treatment is a consensus scenario across three models (COSMO-CLM (Rockel et al., 2008), REMO (Jacob and Podzun, 1997) and RCAO (Döscher et al., 2002)) of climate change in Central Germany for the years between 2070 and 2100. For this, future climate plots (Supplementary Figure S2) are equipped with mobile shelters and side panels as well as an irrigation system, and the roofs are controlled by a rain sensor. Precipitation is reduced by ∼20% in summer months and increased by ∼10% in spring and autumn. The shelters and panels automatically close from sundown to sunrise to increase temperature over all seasons of the year by ∼2°C. More details regarding the GCEF design have been published elsewhere (Schädler et al., 2019).

We performed our analyses in the 10 plots of the extensively managed grassland ecosystem subjected to the future climate scenario (5 plots) in comparison with the plots of ambient climate conditions (5 plots). The meadows are managed by moderate mowing (2–3 times per year) without application of herbicides or fertilizers. The vegetation consists of 56 plant species: 14 grass, 10 legumes, and 32 other herbs (Supplementary Table S1). The experiment was conducted on mid of July 2018 (summer), which was corresponding with the highest effect of the manipulated future climate treatment on soil ecosystem function (plant residues decomposition) at the GCEF (Yin et al., 2019).

### In-situ Soil Bromodeoxyuridine Labeling, DNA Extraction, and BrdU-Labeled DNA Immunocapture

At each plot, three healthy plants of *T. pratense* L. (Red clover) were selected. In-situ soil bromodeoxyuridine (BrdU) labeling was done by injection of 10 ml of 10 mM BrdU in the rooting domain of the soil followed by an incubation time of 24 h, during which the plants were maintained under the surrounding field conditions. The rhizosphere soil samples from the three plants of each plot were then merged into a composite sample. Only active, replicating cells are able to incorporate the BrdU during DNA synthesis, labeling actively growing microorganisms. DNA was extracted from the BrdU-treated rhizosphere soils using a DNeasy PowerSoil kit^TM^ (Qiagen Inc., Valencia, CA, United States) according to the manufacturer’s instructions. Further, we refer to this DNA as “total DNA” as it included all types of genomic DNA (dead cells, dormant cells, in metabolically active, and in replicating cells). BrdU-labeled DNA was isolated from the “total DNA” by an immunocapture approach (Mcmahon et al., 2011) and represents the propagating microbes. Briefly, for each sample, 2 μL monoclonal BrdU antibodies (1 mg μL^–1^ mouse anti-BrdU, clone BU-33, Sigma-Aldrich) was mixed with 18 mL denatured herring sperm DNA [1.25 mg mL^–1^ in phosphate buffer saline (PBS), Promega] and incubated for 45 min at 30°C to form antibody-herring sperm DNA complex. Denatured sample DNA (25 μL ∼200 ng DNA + 10 μL PBS) was then added to antibody-herring sperm DNA complex and incubated for 30 min at 30°C to capture BrdU-labeled DNA. After incubation, the mixture was added to 6.26 μL aliquots of washed Dynabeads^TM^ goat anti-mouse IgG (Invitrogen) in PBS–bovine serum albumin solution (PBS–BSA) and incubated under slow rotation (10 rpm) for 35 min. The Dynabead complex (Dynabead-BrdU antibodies-BrdU-labeled DNA) was washed with 100 ml PBS–BSA eight times by adding the wash solution and trapping the complex with a magnetic particle concentrator (Dynal). BrdU-labeled DNA was released from the washed Dynabeads by adding 25 μl BrdU solution (1.7 mM in PBS–BSA) then incubated under slow rotation for 35 min. The BrdU-DNA was separated from the Dynabeads by using a magnetic particle concentrator, and this DNA is referred to as “active DNA.”

### PCR and Illumina MiSeq Sequencing

Both “total DNA” and “active DNA” were subjected to PCR. The V3–V4 region of the bacterial 16S rRNA was amplified using the forward primer BAC341F (5’-CCTACGGGNGGCWGCAG-3’) (Klindworth et al., 2013) and the reverse primer BAC785R (3’-GACTACHVGGGTATCTAAKCC-5’) (Klindworth et al., 2013), while the internal transcribed spacer region of the fungal internal transcribed spacer region (ITS2) was amplified using the forward primer ITS3 (5’-GCATCGATGAAGAACGCAGC-3’) (White et al., 1990) and the reverse primer ITS4 (5’-TCCTC CGCTTATTGATATGC-3’) (White et al., 1990). Amplification was done in a two-step process. The forward primer of the first PCR was constructed of the Illumina i5 sequencing primer (5’-TCGTCGGCAGCGTCAGATGTG TATAAGA GACAG-3’) and the forward sequence. The reverse primer was constructed with the Illumina i7 sequencing primer (5’-GTCTCGTGGG CTCGGAGATGTGTATAAGAGACAG-3’) and the specific reverse primer. Amplifications were performed in 25 μl reactions with Qiagen HotStar Hi Fidelity Polymerase Kit (Qiagen Inc.), 1 μl of each 5 μM primer, and 1 μl of template. Reactions were performed on ABI Veriti thermocyclers (Applied Biosytems, Carlsbad, CA, United States). Amplification conditions were as follows: 95°C for 5 min, then 35 cycles of 94°C for 15 s, 54°C for 60 s, 72°C for 1 min, followed by one cycle of 72°C for 10 min and 4°C hold. Products from the first stage amplification were added to a second PCR based on qualitatively determine concentrations. During the second PCR, dual indexes were attached using the Nextera XT Index Kit with the same amplification conditions as the first stage, except for 10 cycles. Amplification products were visualized with eGels (Life Technologies, Grand Island, NY, United States) as explained by the manufacturer. Products were then pooled equimolar and each pool was size selected in two rounds using Agencourt AMPure XP (BeckmanCoulter, Indianapolis, IN, United States) in a 0.75 ratio for both rounds. Size selected pools were then quantified using the Quibit 2.0 fluorometer (Life Technologies). Sequencing was performed using MiSeq (Illumina, Inc., San Diego, CA, United States) 2 × 300 bp paired-end strategy according to manufacturer’s manual.

### Bioinformatics

The sequences corresponding to the forward and reverse primers were trimmed from the demultiplexed raw reads using cutadapt (Martin, 2011). Merging of the pair-end raw reads of bacterial and fungal datasets was done using the simple Bayesian algorithm with a threshold of 0.6 and a minimum overlap of 20 nucleotides as implemented in PANDAseq (Masella et al., 2012). All the assembled reads were filtered for high-quality sequence reads (minimum sequence length 390 nucleotides for bacteria, and 120 nucleotides for fungi, maximum sequence length 520 nucleotides for bacteria and 580 nucleotides for fungi, minimum average Phred score of 25, and maximum length of 20 homopolymers in the sequence and without ambiguous nucleotides). Potential chimeras were removed using UCHIME (Edgar et al., 2011) as implemented in MOTHUR (Schloss et al., 2009). High-quality reads were clustered into operational taxonomic units (OTUs) using cd-hit-est 4.6.2 (Fu et al., 2012) at a threshold of 97% pairwise similarity. Bacterial 16S rRNA gene OTU representative sequences were assigned against the SILVA v132 reference sequence database (Quast et al., 2013) to obtain respective OTU tables. Fungal ITS representative sequences were assigned against UNITE v7 sequence database (Kõljalg et al., 2013) using the Bayesian classifier as implemented in MOTHUR (Schloss et al., 2009). Singletons and doubletons OTUs which might have originated from sequencing errors were removed from the datasets. The sequences that were classified as “Cyanobacteria,” “Chloroplast,” or “Mitochondria” or were not classified at the kingdom level were removed from the datasets. Ecological and metabolic functions were predicted for detected bacterial OTUs using FAPROTAX (Louca et al., 2016), the functional Annotation tool of Prokaryotic Taxa v.1.1 and with FUNGuild (Nguyen et al., 2016) for fungi. FAPROTAX is a database providing metabolic or ecological function of prokaryotic clade. The functions of each individual prokaryotic taxa were annotated using literature on cultivable strains. FUNGuild, is a typical functional prediction tool that is used to taxonomically parse fungal OTUs by ecological guild independent of sequencing method.

### Soil Enzyme Analysis

Activities of three hydrolytic enzymes were measured in the same rhizosphere soil samples obtained from each plot. The selected enzymes are involved in the breakdown of cellulose (β-glucosidase), chitin and other β-1,4-linked glucosamine polymers (N-acetyl-glucosaminidase) and polyphosphate (acid phosphatase). We used a fluorimetric method (German et al., 2011; Moorhead et al., 2016) based on the release of 4-methylumbelliferone (MUB) as fluorescent dye-conjugated substrates, 4-methylumbelliferyl-β-glucopyranoside (MUB-g) to detect β-glucosidase activity, 4-methylumbelliferyl-N-acetyl-β-glucosaminide (MUB-N) to detect N-acetyl-glucosaminidase, and 4-methylumbelliferylphosphate (MUB-P) to detect acid phosphatase activity. A sample suspension was homogenized using an ultrasonic bath for 1 min and incubated with the MUB-substrate at room temperature for 1 h. The reaction was terminated by adding 50 μl of 1M NaOH. Fluorescence was measured in microplates at an excitation wavelength of 355 nm and an emission wavelength of 460 nm. Enzyme activities were expressed as MUB release in nmol g^–1^ dry soil hour^–1^.

### Soil Physicochemical Analyses

The rhizosphere soil samples (100–200 g wet weight) from each plot were dried then sieved. The rhizosphere soil pH was measured using WTW Multi 3510 IDS (Weilheim, Germany). Total carbon (TC) and total nitrogen (TN) concentrations in rhizosphere soil were determined by dry combustion at 1000 °C with a CHNS-Elemental Analyzer (Elementar Analysensysteme GmbH, Hanau, Germany) as explained by manufacturer. Then, soil carbon/nitrogen (C/N) ratio was calculated based on TC and TN. Available soil phosphorus was extracted and measured according to Bray 1 method (Gutiérrez Boem et al., 2011). Cations (K^+^, Mg^2+^, Ca^2+^, and Na^+^) in the rhizosphere soil were determined by atomic absorption spectrophotometry according to the manufacturers’ specifications (Hitachi Z 5300, Hitachi—Science & Technology, Japan). Physicochemical properties of soil did not differ significantly between ambient climate and future climate plots (Supplementary Table S2).

### Statistical Analysis

Statistical analyses were performed in PAST program version 2.17c (Hammer et al., 2001) and R environment version 3.6.1 (R-Development-Core-Team, 2019). We normalized the datasets to the minimum number of sequence reads per sample (11,352 and 9,536 sequences reads for bacterial and fungal OTUs, respectively; Supplementary Figure S3) by using the function “rrarefy” from the vegan (Oksanen et al., 2019) package in the R software (R-Development-Core-Team, 2019). Microbial observed OTU richness was calculated and used as a proxy for bacterial and fungal diversity. A permutation multivariate analysis of variance (NPMANOVA) based on Jaccard (presence/absence measures) was performed to test statistical significance among active and total microbial fractions under ambient and future climate regimes. Non-metric multidimensional scaling (NMDS) of the bacterial and fungal communities based on OTU composition was carried out using the Jaccard similarity metric to identify differences between active and total microbial communities under each climate regime. Normal distributions of the data were checked with the Shapiro–Wilk test. Two-way analyses of variance (ANOVA), followed by the Tukey’s *post hoc* test, were used to analyze the effects of microbial fractions (total or active) and climate regime (ambient or future) on the OTU richness on different taxonomic levels (phyla, class, and order) and on the OTU richness of each specific bacterial function and fungal guild. The results considered significant at *p* < 0.05. Pearson correlation was applied on the normally distributed data to test the correlation between microbial OTU richness and respective enzyme activity in soil. To test the correlation between soil physicochemical factors and the different microbial communities (total bacterial community, active bacterial community, total fungal community, and active fungal community) composition, we extracted the information of each community using principal component analysis (PCA). The first, second, and third axis scores of PCA were used to represent the microbial community composition as shown in some previous studies (Ramette, 2007; Purahong et al., 2016). Correlations between the three axis scores of PCA of each bacterial and fungal community and physicochemical parameters or enzyme activities were calculated using Spearman’s product-moment correlation (*p* < 0.05). Correlations between OTUs richness of specific bacterial functions and fungal guilds were calculated using Spearman’s correlation, and Benjamini-Hochberg FDR multiple test correction was applied. The hierarchical structure of taxonomic classification of the detected bacterial and fungal OTUs was presented by a heat-tree (based on relative abundance and presence/absence data) created by metacoder (Foster et al., 2017) package in R software.

## Results

### Richness of Active and Total Rhizosphere Microbiomes Under Climate Regimes

**FIGURE 1:**
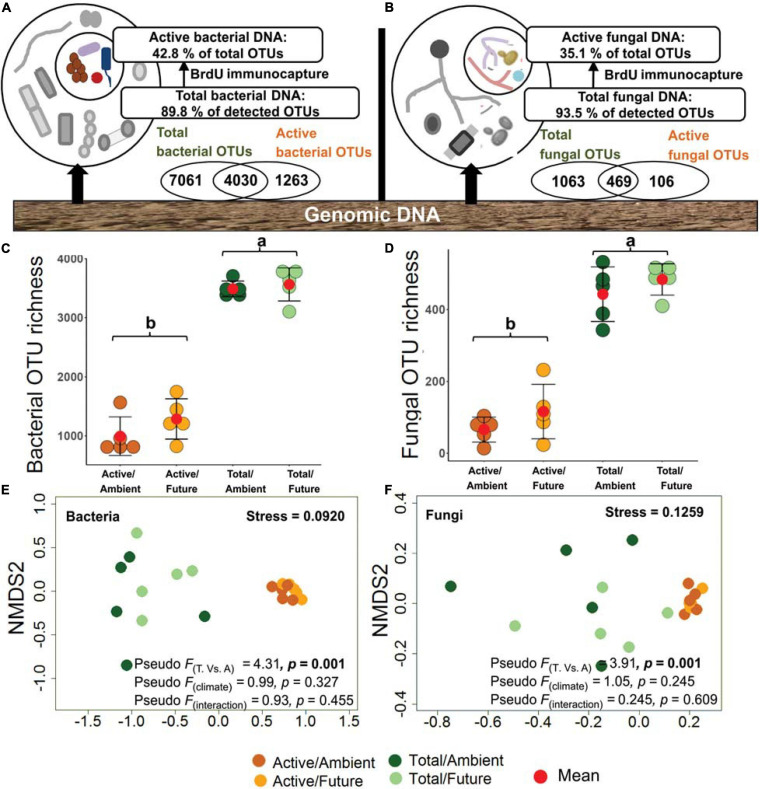
Richness and community composition of active (bromodeoxyuridine-labeled immunocaptured DNA) and total microbial communities. **(A)** 42.8% of bacterial and **(B)** 34.1% of fungal OTUs were representing the active community in the rhizosphere soil. **(C)** Total bacterial OTU richness (3,525 ± 63 OTUs, mean ± SE) was significantly higher (*p* < 0.001) than that of the active community (1,140 ± 110 OTUs). **(D)** OTU richness of total fungal community (463 ± 20 OTUs) was significantly higher (*p* < 0.001) as compared with the active community (91 ± 20 OTUs). Values with the same letter did not differ significantly according to Tukey’s HSD tests. Nonmetric multidimensional scaling (NMDS) ordination plots of active and total **(E)** bacterial and **(F)** fungal community composition under ambient and climate regimes; the plots indicate Jaccard dissimilarity between samples based on presence/absence data; the plots are supported by the results of two-way NPMANOVA. T, total community; A, active community.

Within the bacterial communities, 12,354 OTUs were detected with 4,030 OTUs (32.6%) overlapping between total and active (BrdU-labeled) communities and 1,263 OTUs (10.2%) unique to the active community (Figure 1A). For the fungal community, 1,638 OTUs were detected with 469 OTUs (28.6%) overlapping between total and active communities and 106 OTUs (6.5%) unique to the active community (Figure 1B). Unique active OTUs were the low abundant active microbial communities that were masked by highly abundant inactive community. According to the observed OTU richness of both active and total microbial fractions, we estimated the ratio of the active fraction within the total microbial community to 42.8% for bacteria and 35.1% for fungi in rhizosphere soil (Figures 1A,B and Supplementary Figure S4). The bacterial and fungal OTU richness were used as proxy for microbial diversity in respective active and total communities. As expected, OTU richness of total bacteria and total fungi were significantly higher than those of the active communities (two-way ANOVA, *p* < 0.001), whereby both bacterial and fungal OTU richness did not differ significantly under ambient and future climate regimes (Figures 1C,D).

To test the relation between microbial abundances in active and total community, the relative abundance of “active DNA” sequencing reads (of rarefied OTUs) were correlated to the relative abundance of “total DNA” sequencing reads of the same sample. The Spearman’s rank correlation between the two the sets of relative abundance values, of each sample separately, showed non-significant (ρ = 0.025, *p* > 0.05) or weak (ρ = 0.09 – 0.41, *p* < 0.05) correlations (Supplementary Figure S5) indicating that the presence of microbes in the rhizosphere soil does not imply their activity.

### Compositions of Active and Total Microbial Communities Under Ambient and Future Climate Regimes

Microbial community compositions were analyzed at the OTU level. BrdU-labeled active microbial communities were distinct from the total communities. NMDS ordination based on Jaccard distances grouped all DNA-labeled samples (active community) away from non-labeled samples (total community) (Figures 1E,F). This was confirmed by the two-way NPMANOVA that showed statistically significant differences in community composition between total and active soil microbial fractions in both bacteria (*F* = 4.31, *p* = 0.001) and fungi (*F* = 3.91, *p* = 0.001). However, no significant difference between communities under ambient and future climate regimes was detected (*p* > 0.05) (Figures 1E,F).

**FIGURE 2:**
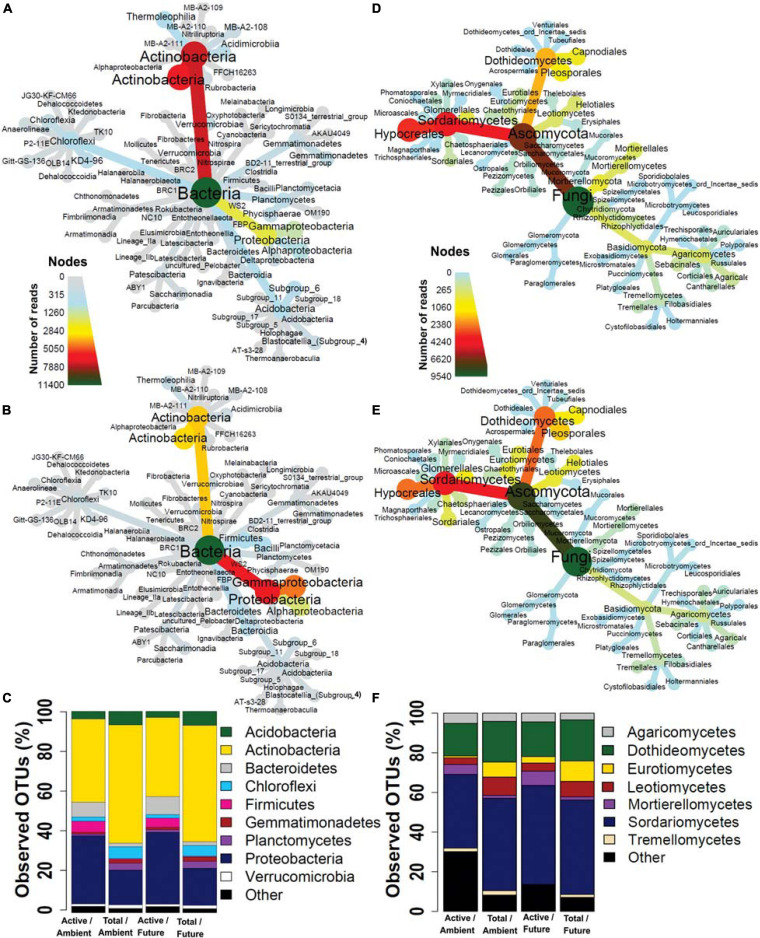
Taxonomic composition of **(A)** total and **(B)** active bacteria and **(D)** total and **(E)** active fungi represented by heat trees. Each node of the heat tree represents a taxon used to classify OTUs, and the edges determine where it fits in the overall taxonomic hierarchy. The color scale of the nodes and edges represents the sequences reads abundance. Microbial community structures at **(C)** phylum level in bacteria and **(F)** class level in fungi. The bar plot shows the OTU richness of the active and total microbial community under the ambient and future climate regimes.

We examined the taxonomic composition of active and total microbial communities in the rhizosphere soil. A total of 24 bacterial (Figures 2A,B and Supplementary Table S3) and 6 fungal phyla (Figures 2D,E and Supplementary Table S4) were retrieved from all datasets but with different relative abundances between active and total community compositions. Unique active microbial taxa represented by low-abundance reads in rhizosphere soil were retrieved by BrdU incorporation (Supplementary Figure S6).

In terms of OTU richness, in the active fraction, the most frequently detected phyla were *Actinobacteria* (41.2% of OTUs), *Proteobacteria* (34.2%), *Bacteroidetes* (8.3%), *Firmicutes* (4.7%), *Acidobacteria* (3.5%), and *Chloroflexi* (2%) (Figure 2C). The most frequently detected phyla in the total community were *Actinobacteria* (58.8%), *Proteobacteria* (17.9%), *Acidobacteria* (6.9%), *Chloroflexi* (5.7%), *Planctomycetes* (3.5%), *Gemmatimonadetes* (1.9%), and *Verrucomicrobia* (1.7%) (Figure 2C). Two-way ANOVA showed that OTU richness of *Proteobacteria*, *Firmicutes*, and *Bacteroidetes* were significantly higher (*p* < 0.05) in the active than in the total communities while significantly more *Actinobacteria*, *Acidobacteria*, *Chloroflexi*, *Gemmatimonadetes*, *Planctomycetes*, and *Verrucomicrobia* were present in the total community than in active community. The climate regime had no significant effect on any of the detected phyla. 18 fungal classes were detected from all fungal communities where *Ascomycota* was the most frequently detected phylum accounting for 72.5 and 88.2% of detected OTUs in active and total community, respectively, followed by *Basidiomycota* accounting for 7.6 and 6.4% of detected OTUs in active and total community, respectively (Figure 2F). *Mortierellomycetes* and *Agaricomycetes* were represented by significantly higher OTU richness in the active community than in the total community. On the other hand, *Dothideomycetes*, *Eurotiomycetes*, *Leotiomycetes*, *Pezizomycetes*, *Tremellomycetes*, and *Glomeromycetes* were represented by significantly more OTUs in the total community than in the active one. Significantly more *Eurotiomycetes* and *Pezizomycetes* OTUs were present under future than ambient climate regime, while significantly more *Sordariomycetes* OTUs were present under ambient climate than future climate regime (two-way ANOVA, *p* < 0.05). Other taxonomic levels are illustrated in Supplementary Figures S7–S18.

### Soil Physicochemical Factors Corresponding With Microbial Community Compositions

The Spearman’s rank correlation between soil parameters, and microbial communities were used to determine which factors shape active and total microbial community compositions. Total and active bacterial and fungal communities correlated with completely different soil physicochemical factors (Table 1). C/N, N and pH were found to be significantly correlated with the total bacterial communities (*p <* 0.05). The active bacterial community was correlated only with Ca^2+^ (*p <* 0.05). Significant correlation of soil organic matter and moisture were detected with the total fungal community (*p <* 0.05). Active fungal community was correlated with both P and Ca^2+^ (*p <* 0.05).

**TABLE 1:** Spearman’s rank correlations between the community composition of different microbial communities and soil physicochemical factors and enzymes activity.

Community composition	Soil parameters											Soil function		
	pH	OM	P	CEC	K	Na	Ca	Mg	C/N ratio	N%	Soil moisture	β-gluc	Pho	Nag
Total bacteria	0.697*	***0.600***	0.224	0.198	0.055	0.200	***0.564***	0.188	0.806*	0.718*	0.103	0.055	0.382	0.224
Active bacteria	***0.261***	0.224	***0.588***	0.471	0.030	0.164	0.661*	***0.582***	0.103	0.576	0.321	***0.588***	0.067	0.648*
Total fungi	0.455	0.721*	0.285	***0.564***	0.176	0.103	0.382	0.194	0.430	0.235	0.636*	0.370	0.745*	0.673*
Active fungi	0.467	0.491	0.673*	***0.595***	0.261	***0.588***	0.661*	***0.594***	0.188	0.254	0.261	0.321	***0.600***	0.079

Significant ρ values are indicated in bold* (*p* < 0.05), and marginal significant are indicated in italic (*p* < 0.1). β-gluc, β-glucosidase; Pho, acid phosphatase; Nag, N-acetyl-glucosaminidase.

### Functional Assignment of Active and Total Rhizosphere Microbial Community Compositions

**FIGURE 3:**
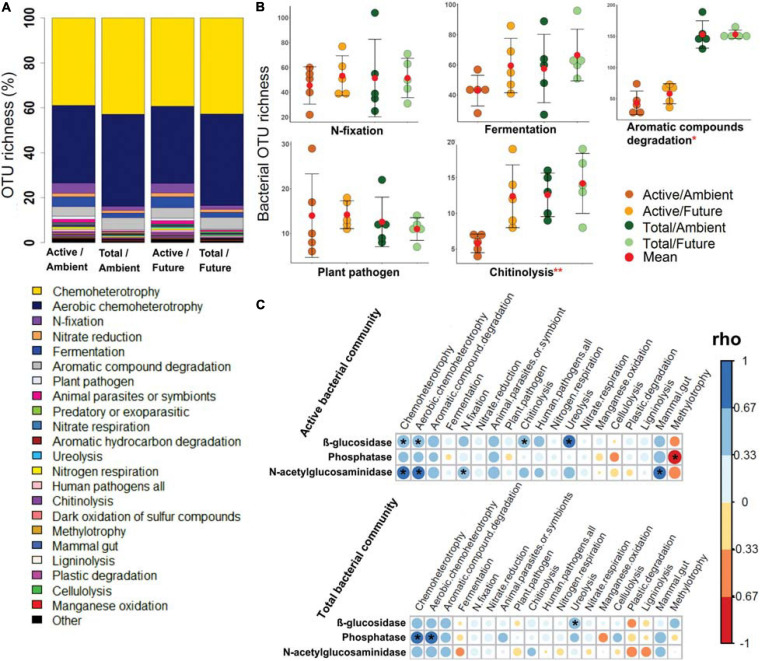
The potential bacterial functions based on FAPROTAX database. **(A)** Bar plot shows a comparison of predicted functions between active and total bacterial communities under ambient and future climate regimes based on the percentage of observed OTUs in each treatment. **(B)** OTU richness of five of highly abundant functions. *Significant difference of functional richness between metabolically active and total community (*p* < 0.05, two-way ANOVA test). **Significant difference of functional richness between metabolically active and total community and a significant influence of climate regime on the functional richness (*p* < 0.05, two-way ANOVA test). **(C)** Spearman’s rank correlation (rho) between the OTU richness of detected functions (in active and total community composition) and the measured enzyme activities in the rhizosphere soil. *Significant correlations (*p* < 0.05) between the two factors are indicated by asterisks.

All predicted bacterial trophic modes and functions were present among the members of the active as well as the total community compositions under ambient and future climate regimes (Figure 3A). The OTU richness of the specific bacterial functions (e.g., N-fixation, fermentation, plant pathogen, nitrate respiration, ligninolysis, cellulysis, methylotrophy, and animal parasites) was not statistically different between active and total communities (two-way ANOVA, *p* > 0.05) (Figure 3B and Supplementary Figure S19). We also found that the future climate had almost no impact on the predicted metabolic functions, except for chitinolysis and ureolysis bacteria as they showed a significantly higher OTU richness (two-way ANOVA, *p* < 0.05) under the future climate than ambient climate regime.

**FIGURE 4:**
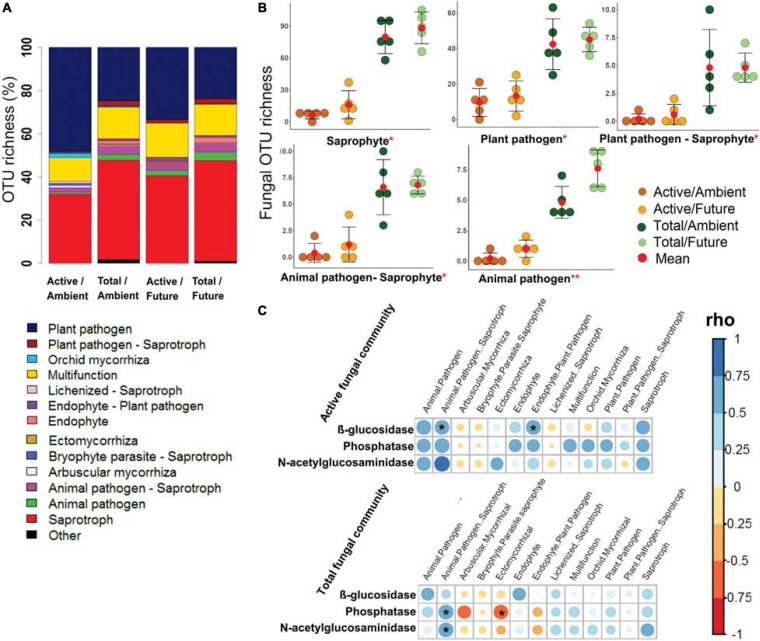
The predicted fungal guilds based on the FUNguild database. **(A)** Bar plot shows a comparison of ecological guilds between active and total fungal communities under ambient and future climate regimes based on the percentage of observed OTUs in each treatment. **(B)** Richness of five of highly abundant specific guilds at OTU level. *Significant difference of functional richness between metabolically active and total community (*p* < 0.05, two-way ANOVA test). **Significant difference of functional richness between metabolically active and total community and a significant influence of climate regime on the functional richness (*p* < 0.05, two-way ANOVA test). **(C)** Spearman’s rank correlation (rho) between the OTU richness of detected functions (in active and total community composition) and the measured enzyme activities in the rhizosphere soil. *Significant correlations (*p* < 0.05) between the two factors are indicated by asterisk.

The OTU richness of assigned fungal guilds revealed that the active fungal community was governed by plant pathogens (40.7–24.1% of OTUs in active and total community composition) and saprotrophs (36.3–46.4% of OTUs) (Figure 4A). The OTU richness of the major detected guilds were significantly higher (two-way ANOVA, *p* < 0.05) in the total community than in the active community (Figure 4B and Supplementary Figure S19). The effect of climate regime was detected only in case of animal pathogens where the OTU richness was significantly higher under the future climate than ambient climate regime.

### Relationship Between Biodiversity and Ecosystem Functioning

No effects of the climate regime on activities of β-glucosidase and acid phosphatase were observed. In contrast, the activity of N-acetyl-glucosaminidase (t-test, *p* = 0.02) was significantly higher under the future climate regime (Supplementary Figure S20). We investigated the relationships between the studied ecosystem function (extracellular enzymes) and (i) active and total microbial diversity (OTU richness), (ii) OTU richness of specific bacterial functional groups and fungal guilds of the active and total communities and (iii) the community composition of active and total bacteria and fungi.

**FIGURE 5:**
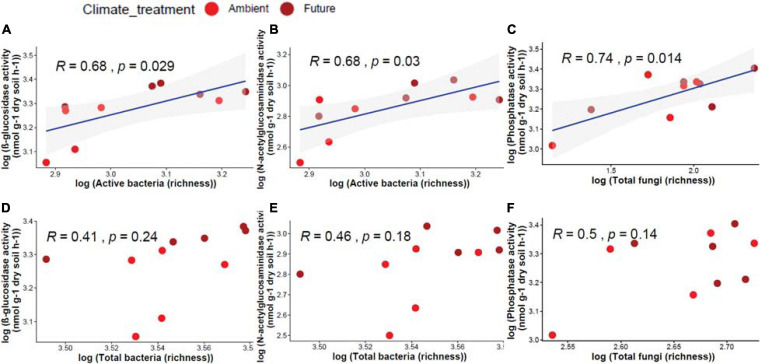
Pearson correlation between OTU richness of different microbial communities and the measured enzyme activity in soil showing a significant positive correlation between active bacteria and **(A)** β-glucosidase, **(B)** N-acetyl-glucosaminidase, and **(C)** active fungi and phosphatase activity. Non-significant correlations between total bacteria and **(D)** β-glucosidase, **(E)** N-acetyl-glucosaminidase activity, and **(F)** total fungi and acid phosphatase activity.

Active and total microbial community richness and enzyme activity were correlated. We found that active bacterial OTU richness correlated significantly positively with β-glucosidase (*R* = 0.68, *p* = 0.029) and N-acetyl-glucosaminidase (*R* = 0.68, *p* = 0.03) activities (Figures 5A,B), while active fungal OTU richness correlated significantly positively with acid phosphatase (*R* = 0.74, *p* = 0.014) activity (Figure 5C). On the other hand, total bacterial and fungal communities had no significant correlation with any enzyme activities (Figures 5D–F).

Further analyses revealed significant positive correlations of the OTU richness of specific active bacterial and fungal functional groups with different enzymes activities. For instance, chemoheterotrophic and aerobic chemoheterotrophic active community richness correlated with β-glucosidase and N-acetyl-glucosaminidase activity, atmospheric N-fixing active groups correlated positively with N-acetyl-glucosaminidase activity, while ureolysis and chitinolysis correlated positively with β-glucosidase activity (Figure 3C). Furthermore, active saprotrophic, endophytic, and plant pathogenic fungal richness positively correlated with β-glucosidase enzyme activity (Figure 4C). Few specific ecological functional groups of total bacterial and fungal communities showed significant correlations with soil enzyme activities (Figures 3C, 4C).

N-acetyl-glucosaminidase correlated with the community compositions of active bacterial (*p* = 0.04) and total fungal communities (*p* = 0.03) (Table 1). Active bacteria were also marginally significantly correlated with β-glucosidase activities (*p* = 0.07). Acid phosphatase was correlated with both total (*p* < 0.05) and active (*p* = 0.07, marginal significant) fungal communities (Table 1).

## Discussion

### Estimation of the Active Microbial Fraction in the Rhizosphere Soil Using BrdU-Immunocapture Approach

Several studies have already estimated the proportion of active soil microorganisms in different ecosystems. The ratio of active microbes in soil based on a microbial cultivation approach and direct microscopic estimations after cell staining revealed that 10–40% (up to 60%) of the total microbial biomass was potentially active microbes (Blagodatskaya and Kuzyakov, 2013). Another study reviewed that approx. 80% of total cell counts as determined by fluorescence *in situ* hybridization (FISH) or staining with CTC, and approx. 50% of OTUs of ribosomal RNA–ribosomal DNA terminal restriction fragment length polymorphism (TRFLP) in bulk soil may be inactive (Lennon and Jones, 2011). In our study, 43 and 35% of the detected bacterial and fungal community richness, respectively, represented active members (based on BrdU immunocapture and Illumina sequencing) in the rhizosphere soil of *Trifolium pratense* of a grassland ecosystem. The majority of bacterial and fungal phyla and ecological functions were represented in the active fraction, indicating that a broad microbial spectrum was capable of BrdU uptake and that its detection by immunocapture technique works. The experiment was performed at the Global Change Experimental Facility, a field infrastructure with a realistic scenarios to compare ecosystem effects on biological systems, so the incubation conditions with BrdU was not artificial, no additional substrates (isotopically labeled) were added, and therefore, the results reflect a realistic image of the soil microbial community in an active status. Despite the general assumption that the active soil community represents a subset of the total soil community, reported results showed that these two fractions are quite independent from each other and that the active community is similar in richness as or even more taxonomically diverse than the total community (Baldrian et al., 2012; Romanowicz et al., 2016; Nawaz et al., 2019). In our approach, the active bacterial and fungal community composition could, however, be considered as a subsets of the total microbial community composition, as we detected a low proportion of active bacterial (10.2%) and fungal (6.5%) OTUs unique to the active fraction. These unique active communities were masked by the high abundant inactive taxa. Application of the BrdU-immunocapture has removed the interference of inactive highly abundant microbes.

### Active and Total Microbial Communities Composition Are Different and Are Shaped by Soil Physicochemical Factors, but Not by Climatic Factors

Similar to previous studies in rhizosphere and bulk soils of other ecosystems (Freedman et al., 2015; Ragot et al., 2016; Cardoso et al., 2017; Li et al., 2019), our results indicated that there are significant differences between the total and the active microbial community compositions. We found that the total community was more diverse than the active one. This could be explained by the fact that rhizosphere is a highly chemically dynamic compartment with tremendous microbial interactions (Singh et al., 2004; Zhalnina et al., 2018). Thus, at any given time only some specific substrates are available and only some microbes with ability to use these substrates have a chance to become active (Vieira et al., 2019). The rest of the microbes may stay inactive until suitable substrates are present in the rhizosphere (Lennon and Jones, 2011). In addition, appropriate conditions (i.e., edaphic, climatic, and biotic factors) may also play very important roles to activate microbes (Li et al., 2015; Birgander et al., 2018).

Our results revealed that total and active microbial communities are shaped by different soil factors. There appears to be no study comparing active and total microbial community in the rhizosphere soil in comparable systems with our study, thus we compare our results to studies in bulk soils. Studies in the bulk soil of temperate grassland and forest ecosystem emphasized that the same environmental factors (soil moisture, pH as well as soil C and N) shaped the total and active bacterial communities (Ragot et al., 2016; Romanowicz et al., 2016). Also, the same environmental factors shaped the total and active fungal community in bulk soil of a forest ecosystem (Romanowicz et al., 2016). We do find that these factors shape the total microbial communities in the rhizosphere soil, but not the active microbial communities (Table 1). We found that the active bacterial communities were correlated with Ca^2+^ while active fungal communities were correlated with Ca^2+^ and P. This could be explained by the importance of these elements for active microorganisms because of their contribution to active physiological processes. Ca^2+^ is involved in a number of bacterial processes such as maintenance of cell structure, motility, adhesion, cell division, gene expression and cell differentiation processes such as sporulation, heterocyst formation, fruiting body development and biofilm formation (Norris et al., 1996; Torrecilla et al., 2004; Das et al., 2014). Also, Ca^2+^ is involved in hyphal tip growth of fungi (Jackson and Heath, 1993). Previous studies reported that Ca^2+^ content in soil influence bacterial community structure (Rezapour, 2013; Xue et al., 2017). Furthermore, P is essential as part of many cellular compounds, such as DNA and the energy carrier adenosine triphosphate (ATP). Available P in our system (extensively managed grassland) is limited as we do not supply any fertilizer, thus P mineralization and assimilation are important processes in the rhizosphere. Members of the active fungal community composition were capable to assimilate the dissolved phosphate in soil (Caldwell, 2005). Similar to these results we found a positive correlation between active fungal community composition and phosphatase activity, indicating that some active fungi hydrolyze P from organic compounds and thereby making P bioavailable in the rhizosphere soil. In this study of a microbial hot spot, we demonstrated that active microbial community composition was very differently organized compared to the total community with no overlapping factors shaping respective community compositions. In less active environments such as bulk soil, the total and active community compositions were different but they were shaped by similar soil physicochemical properties (Ragot et al., 2016; Romanowicz et al., 2016).

At the Global Change Experimental Facility (GCEF), the future climate scenario regime was started in 2014 and included altered precipitation patterns during the year and an increase of mean annual temperatures by ∼2°C (Schädler et al., 2019). Our sampling was performed after four years of climate manipulation, however, our results indicated that both bacterial and fungal communities, except for specific few taxa, have not been influenced by the future climate scenario. Microbial communities may have been resistant to the changing environmental factors, enabled by microbial trait plasticity. Another possibility is that microbial communities showed some resilience and returned to its original composition after being disturbed during the drought periods of summer months (Allison and Martiny, 2008). In addition, it is possible that microbial communities changed at the genomic level that were not visible from 16S and ITS sequencing data. Also, investigation of the microbial extracellular enzymes production revealed mostly a resistance toward the future climate scenario. The recovery of enzymes has been also reported during the rewetting after drought periods (Pohlon et al., 2013). In addition, it is interesting to find one of the most important bacterial function, atmospheric N-fixation, has not been altered by changing climate. *T. pratense* is an efficient atmospheric N fixer plant because of the symbiosis with nitrogen fixing bacteria (Fustec et al., 2010) suggesting that *T. pratense* could be considered as a soil fertility supporting crop in the future.

### Ecosystem Functions Are Linked With Active Rather Than Total Microbial Diversity

The relationship between microbial diversity and ecosystem functioning (BEF) is complex and understanding this elusive link is one of the most demanding scientific challenges (Cardinale et al., 2012). In soil, a large portion of the microbial diversity detected may not contribute to functions at a given point in time, obscuring microbial BEF studies. We found that the studied ecosystem function (enzymes activity) was correlated with active but not total microbial communities.

In addition, the enzymes’ activity was linked with the richness of specific functional classes (metabolic function or functional guilds) of the active community. For instance, we found that the richness of active chemoheterotrophic and aerobic chemoheterotrophic communities, the two dominant trophic modes, positively correlated with β-glucosidase and N-acetyl-glucosaminidase activity. This can be explained by the fact that the bulk of the enzyme activity is contributed by microbes that can be characterized by their high occurrence and large biomass, comparatively higher metabolic activity and larger quantities of secretion of extracellular enzymes into the soil (Joshi et al., 2018). It is also interesting that active bacteria associated with N cycling (N-fixing) were positively linked with activity of N acquisition enzyme (N-acetyl-glucosaminidase). Due to the high activity of N-fixing microbes in our rhizosphere soil, Nitrogen fixed by *T. pratense* microbes is released into the soil mainly through N-containing exudates as well as root decomposition (Thilakarathna et al., 2018). As a result, the N content in the form of NO_3_^–^-N, NH_4_^+^ -N and dissolved organic N is increased in the rhizosphere (Thilakarathna et al., 2018) leading to increase in the activity of N-acetyl-glucosaminidase enzyme (Schleuss et al., 2019). Moreover, richness of active fungi was positively correlated with phosphatase activity. Our study field is characterized by no application of fertilizers, which resulted in limitation of P. We detected many fungal genera capable of solubilization and mineralization of insoluble soil phosphate to release soluble P and making it available to plants (Alori et al., 2017), including *Alternaria, Arthrobotrys, Aspergillus, Cladosporium, Curvularia, Fusarium, Myrothecium, Oidiodendron, Paecilomyces, Saccharomyces, Schwanniomyces, Torula*, and *Trichoderma.*

Our findings thus provide experimental evidence that soil ecosystem function can be reasonably predicted by the overall and function specific richness and the community composition of active microbial community. We performed our study in a short time scale, and hence, large-scale ecological studies are needed to assess our findings, including other ecosystems and time scales.

## Conclusion

Soil, the rich ecosystem, includes numerous and diverse microorganisms. Some microbes were active and responsible for ecosystem function, while other are non-active and may serve as a backup of functional redundant microbes and/or a reservoir of the genetic information. We found that the composition of the active and total microbial community compositions were distinct from each other. Moreover, the active communities were more accurately to reflect the correlation between tested soil function and microbial richness. Furthermore, we provide evidence on the factors shaping active community compositions in the rhizosphere soil, which were totally different from those that shape the total community composition. Finally, our results showed that soil microbes in the rhizosphere of *T. pratense* (both active and inactive portions) were highly adaptable to the future climate changes, and thus, they can provide soil ecosystem functions nowadays and in the future.

## Data Availability Statement

The bacterial 16S and fungal ITS2 raw read sequence datasets were deposited in the National Center for Biotechnology Information (NCBI) Sequence Read Archive (SRA) under bioproject number PRJNA556361.

## Author Contributions

WP, FB, SW, and BT conceived and designed the study. MS was responsible for the coordination of GCEF platform. SW, WP, and BT conceived the field sampling. SW and BT conceived the molecular work. SW, CS, and MN conceived the enzymes analysis. AH-B led the bioinformatics analysis. YW conceived the soil physicochemical analysis. SW and WP analyzed the data. SW wrote the manuscript. FB, MN, WP, AH-B, and MS edited the manuscript. All of the authors reviewed and gave comments and suggestions for the manuscript. All of the authors gave final approval for the manuscript submission.

## Conflict of Interest

The authors declare that the research was conducted in the absence of any commercial or financial relationships that could be construed as a potential conflict of interest.

## Abbreviations

BEF: biodiversity-ecosystem functioning
BrdU: bromodeoxyuridine
OTU: operational taxonomic unit
GCEF: Global Change Experimental Facility.
