# Supplementary material for: Targeting the Active Rhizosphere Microbiome of Trifolium pratense in Grassland Evidences a Stronger-Than-Expected Belowground Biodiversity-Ecosystem Functioning Link
Source: Front Microbiol. 2021 Feb 1;12:629169. doi: 10.3389/fmicb.2021.629169 (PMC7882529; doi:10.3389/fmicb.2021.629169)
Supplement: Supplementary file 3 [file Data_Sheet_1.docx]

Supplementary Material

1. **Supplementary Tables**

**Supplementary Table S1.** The vegetation of the extensively managed grassland plots - Global change experimental facility (GCEF)

**
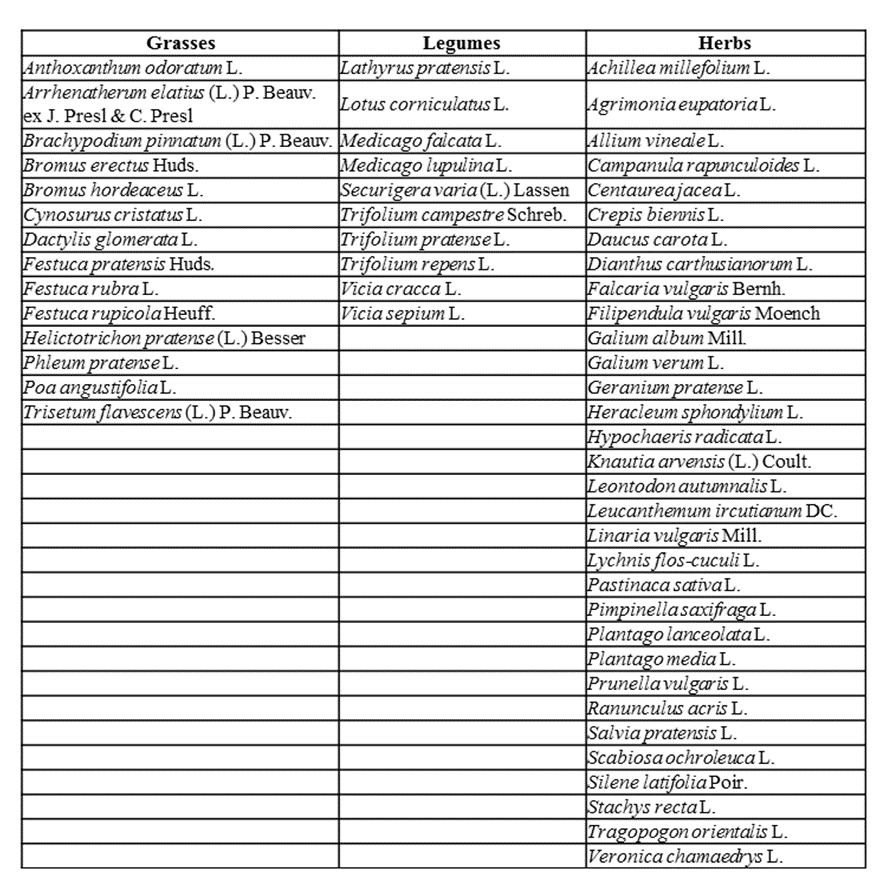
**

**Supplementary Table S2.** Physicochemical properties of rhizosphere soil of ambient and future climate. Values represent mean ± sd. The values did not differ significantly between ambient and future climate soils (t-test, p>0.05).

| Edaphic factor | Ambient climate | future climate |
| --- | --- | --- |
| pH | 6.47±0.18 | 6.53±0.17 |
| Organic Matter (%) | 5.18±0.72 | 4.45±0.41 |
| P (ppm.) | 129.86±18.49 | 124.16±14.33 |
| CEC | 8.64±0.26 | 8.64±0.3 |
| K (m.e/100g soil) | 1.12±0.36 | 1.19±0.21 |
| Na (m.e/100g soil) | 0.43±0.25 | 0.54±0.39 |
| Ca (m.e/100g soil) | 21.08±4.82 | 19.39±3.83 |
| Mg (m.e/100g soil) | 2.4±0.03 | 2.55±0.39 |
| C/N | 11.79±2.25 | 10.25±1.82 |

**Supplementary Table S3.** Sequence read numbers of the bacterial OTUs of active and total community.

**Supplementary Table S4.** Sequence read numbers of the fungal OTUs of active and total community.

1. **Supplementary Figures**

**
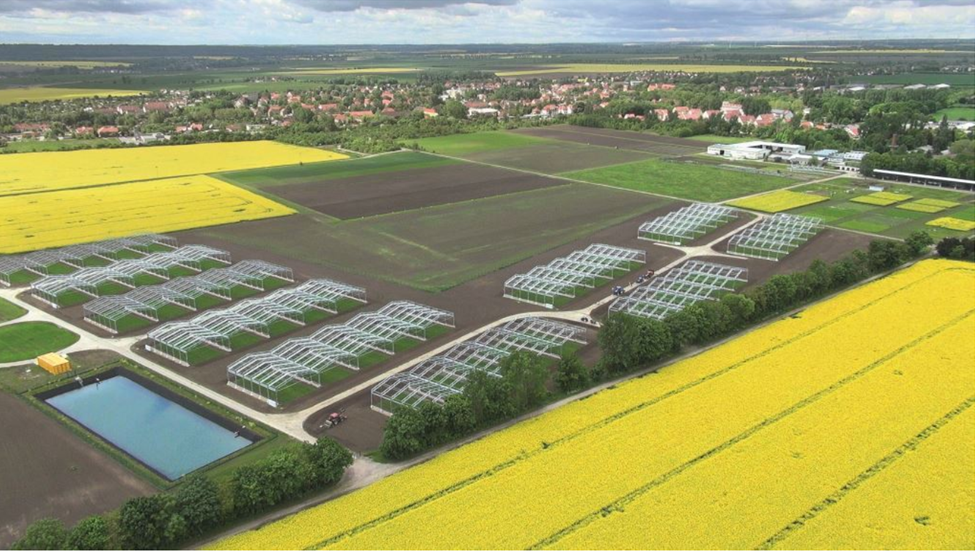
**

**Supplementary Figure S1.** Aerial view for the *Global Change Experimental Facility* (*GCEF*) field research station of the Helmholtz Centre for Environmental Research in Bad Lauchstädt, Saxony-Anhalt, Germany, photo taken by Tricklabor Berlin/Service Drone.


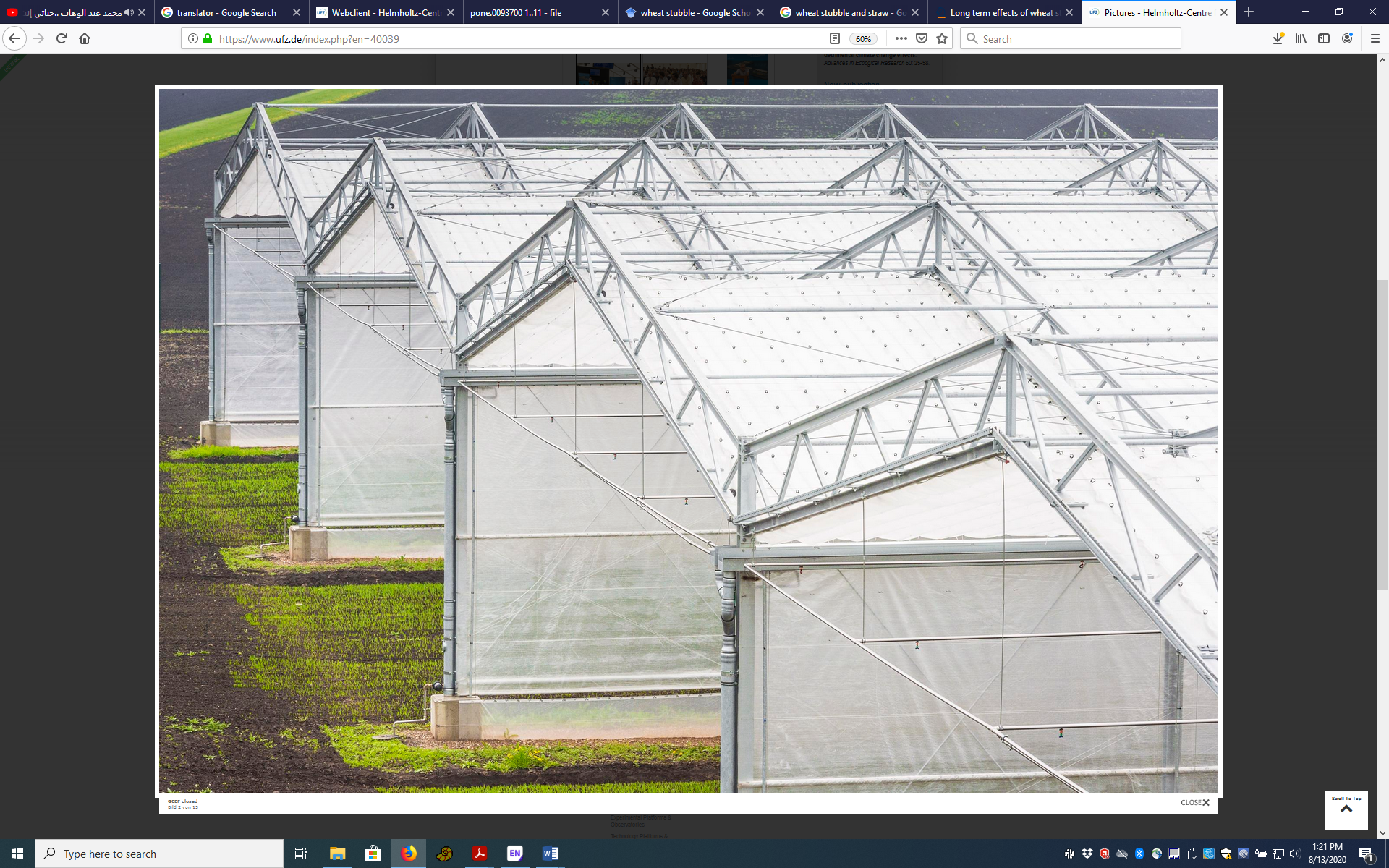


**Supplementary Figure S2.** Closed shelters and panels of the future climate plots of the *GCEF*, photo taken by UFZ/ André Künzelmann


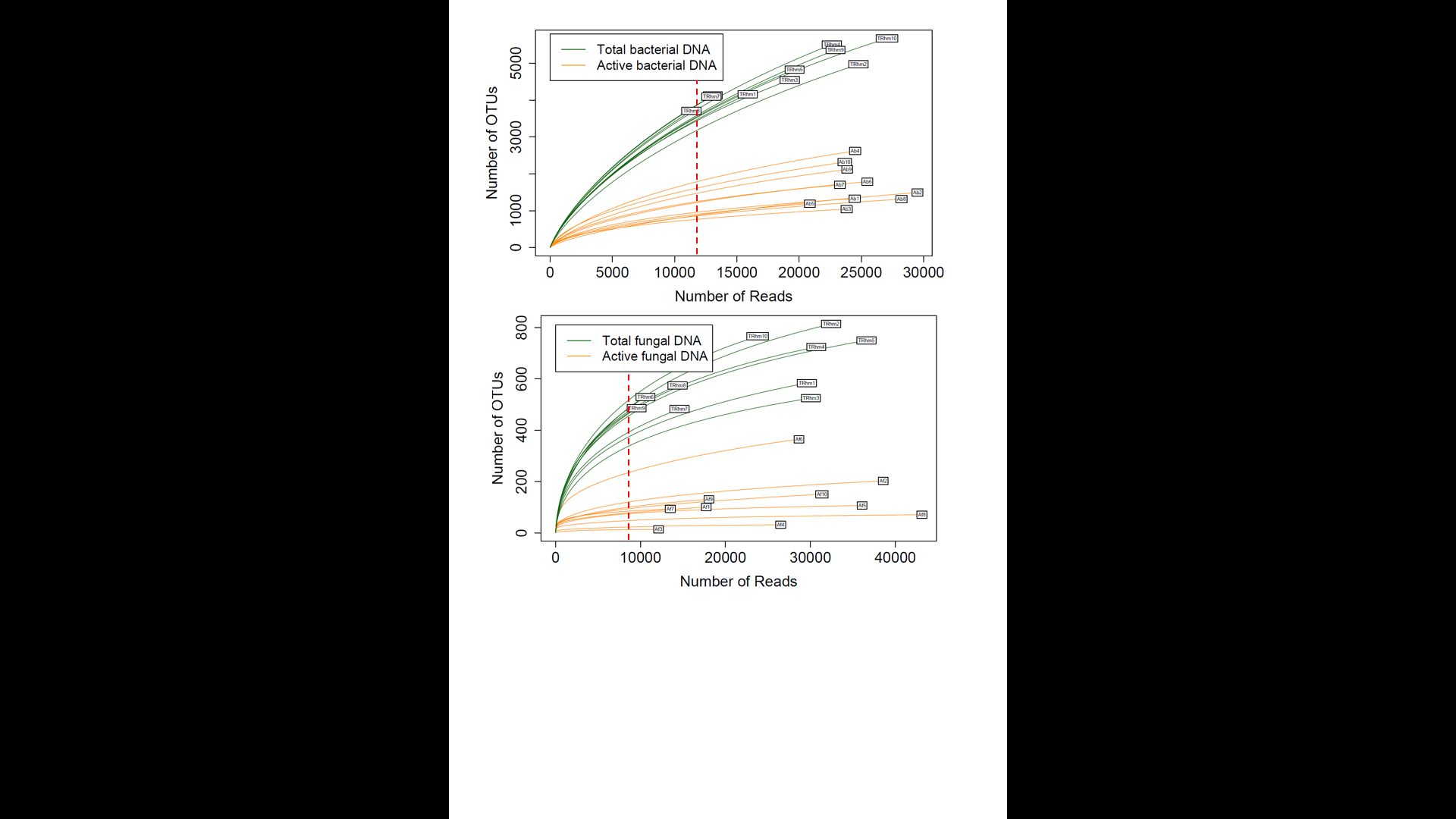


**Supplementary Figure S3.** Rarefaction curves for bacterial communities and fungal communities. OTUs are estimated at a 3% difference level.


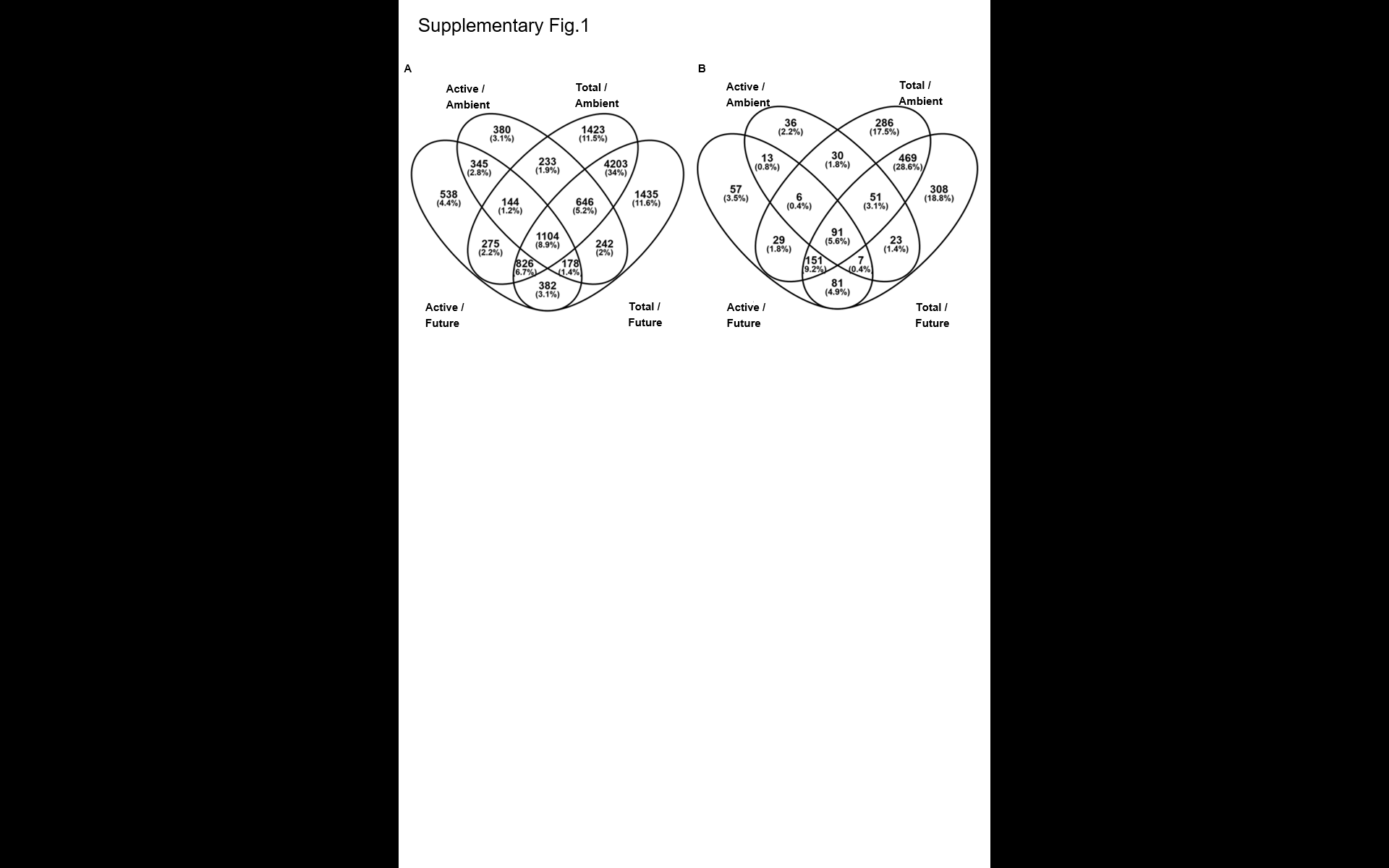


**Supplementary Figure S4.** Venn diagram showing treatment-specific and shared **(A)** bacterial and **(B)** fungal OTUs of active and total microbes across ambient and future climate regimes.


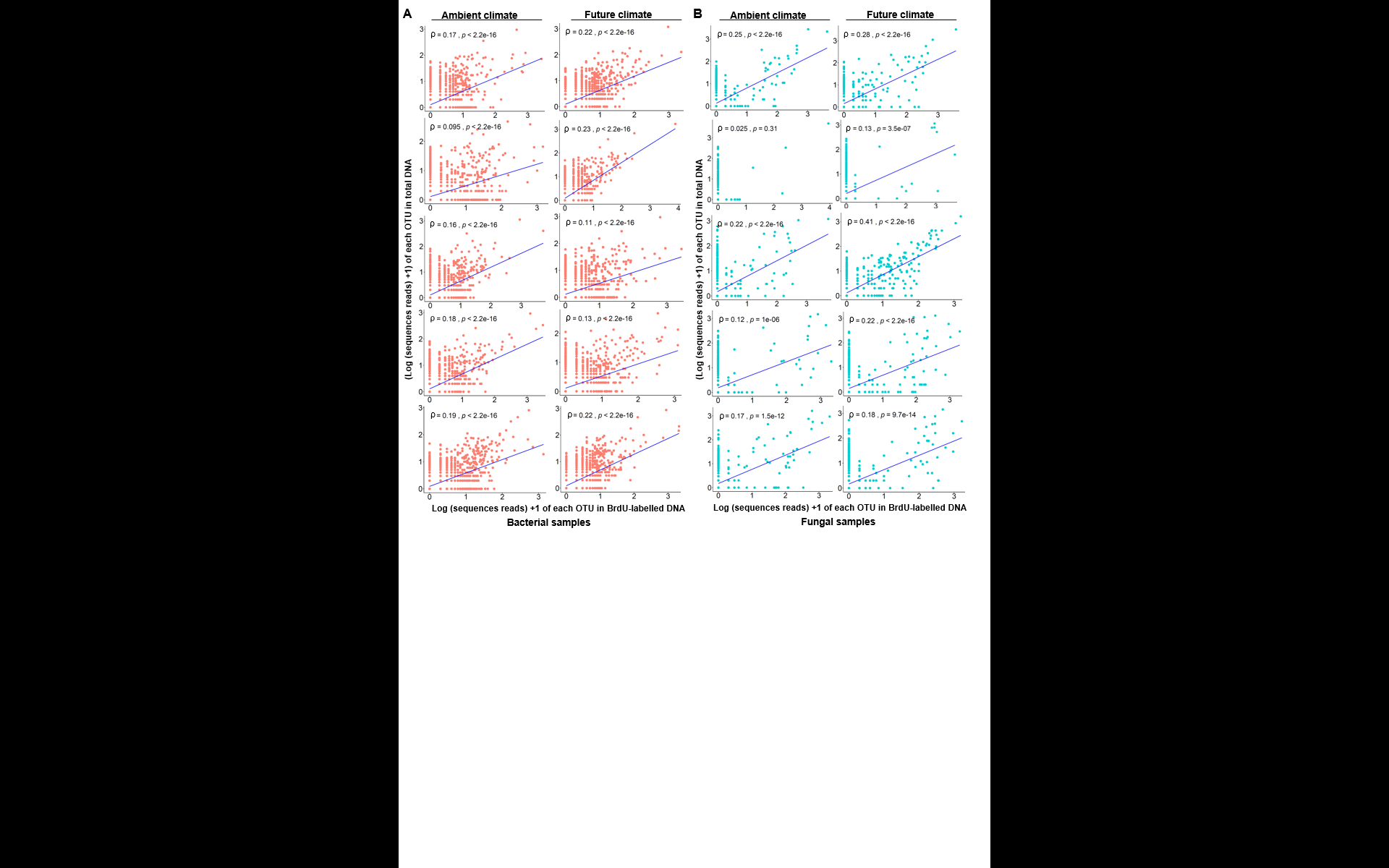


**Supplementary Figure S5.** Spearman’s rank correlation between numbers of sequences reads of each OTU in BrdU-labelled active DNA and number of sequences reads in the corresponding total DNA after rarefaction. **(A)** bacterial OTUs (orange color), **(B)** fungal (blue color) samples.


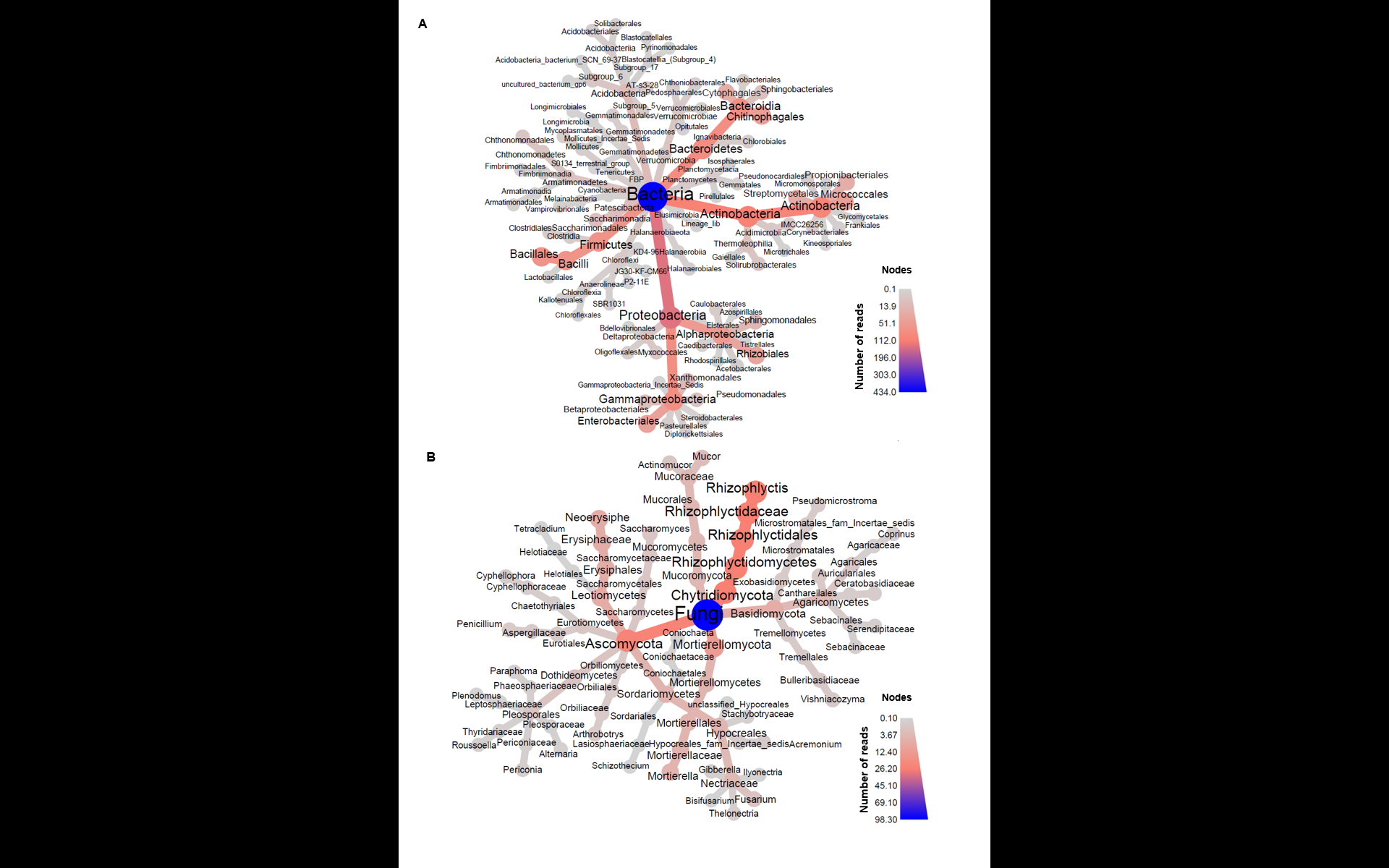


**Supplementary Figure S6.** Relative abundance of unique active taxa retrieved by BrdU-immunocapture incorporation at **(A)** order level in bacteria and **(B)** genus level in fungi.


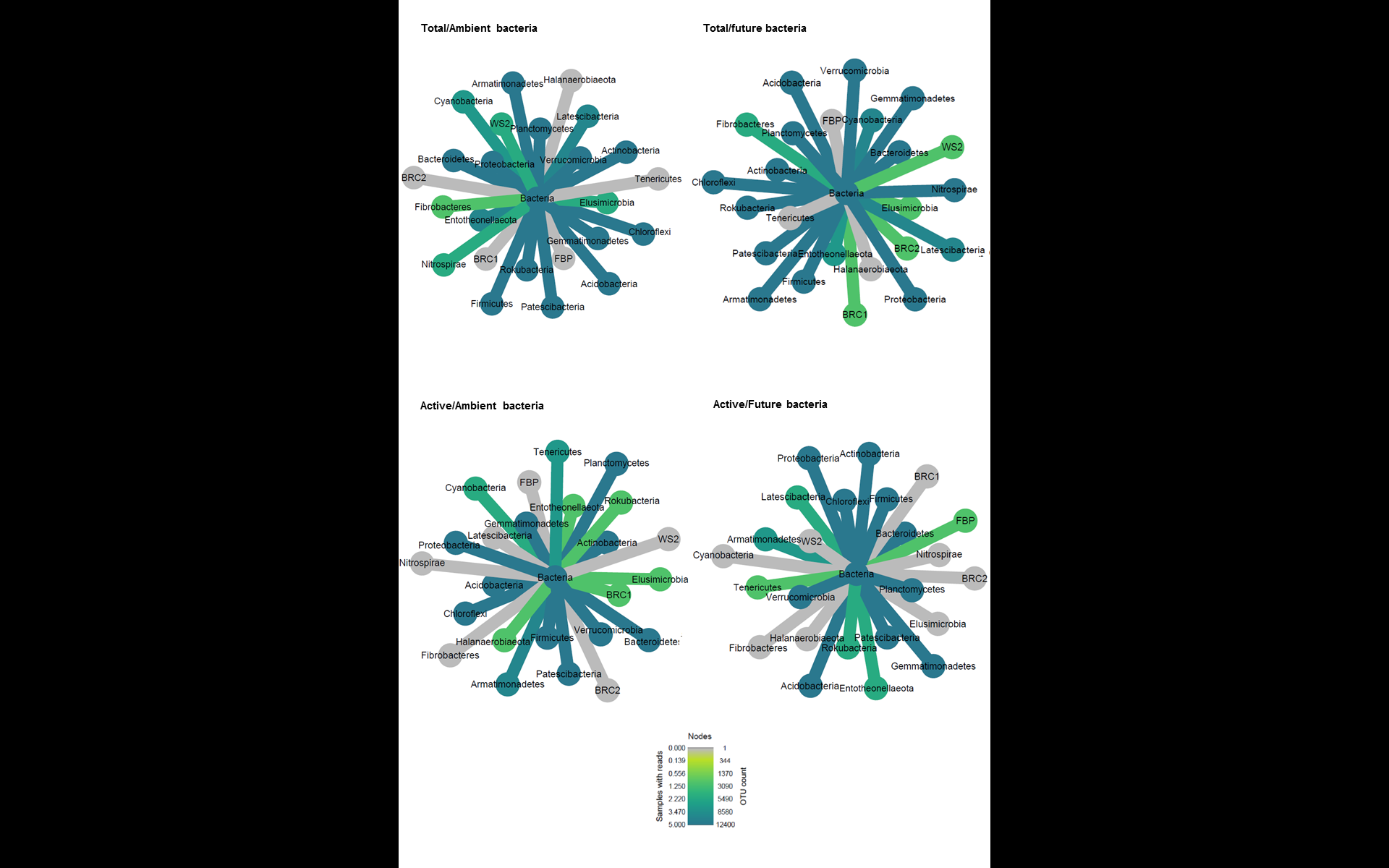


**Supplementary Figure S7.** Community composition of active and total bacteria under ambient and future climate regimes represented by heat tree of overall bacterial microbiome of each microbial fraction, taxa are shown to phylum level.


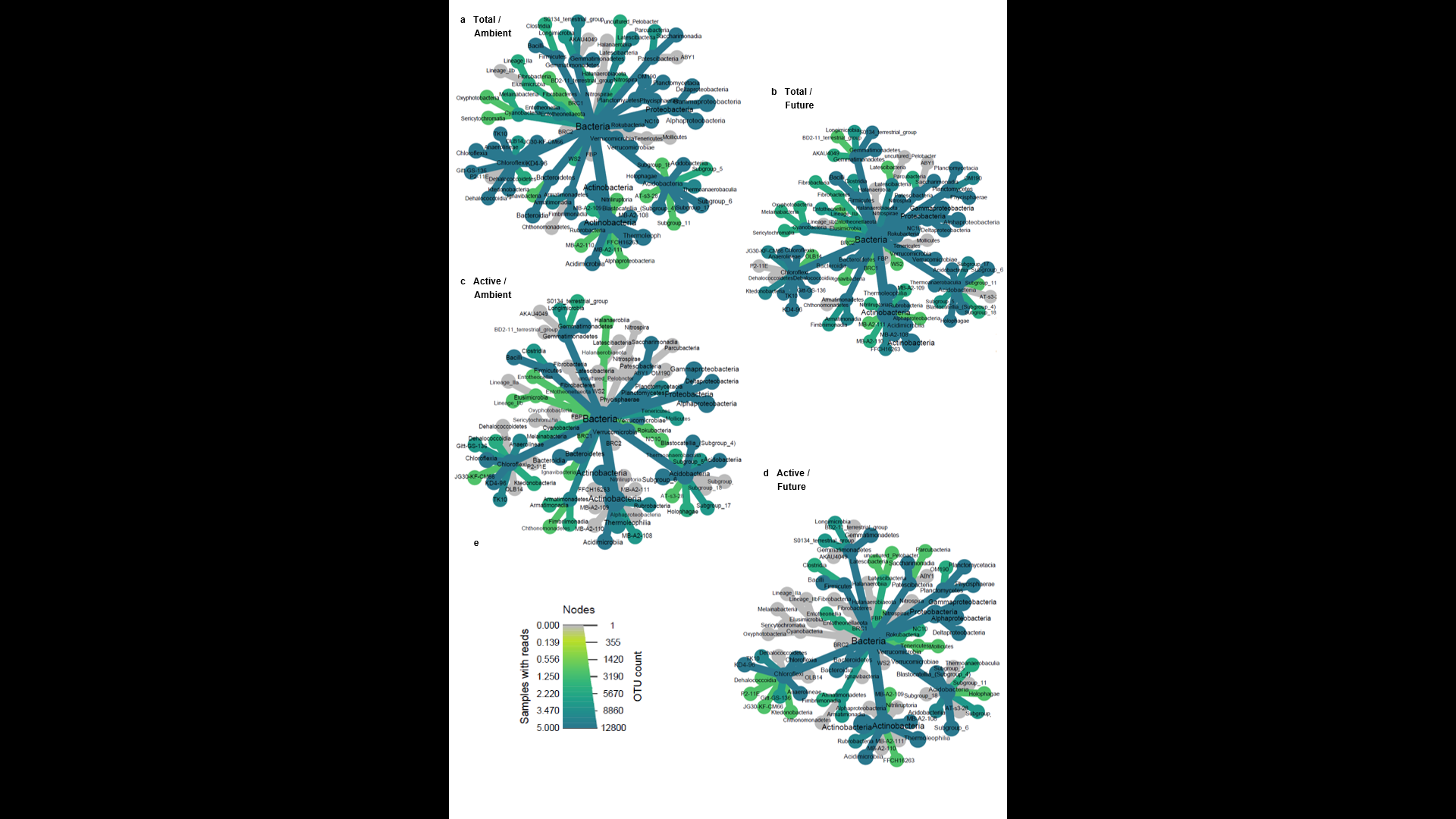


**Supplementary Figure S8.** Community composition of active and total bacteria under ambient and future climate regimes represented by heat tree of overall bacterial microbiome of each microbial fraction, taxa are shown to class level.


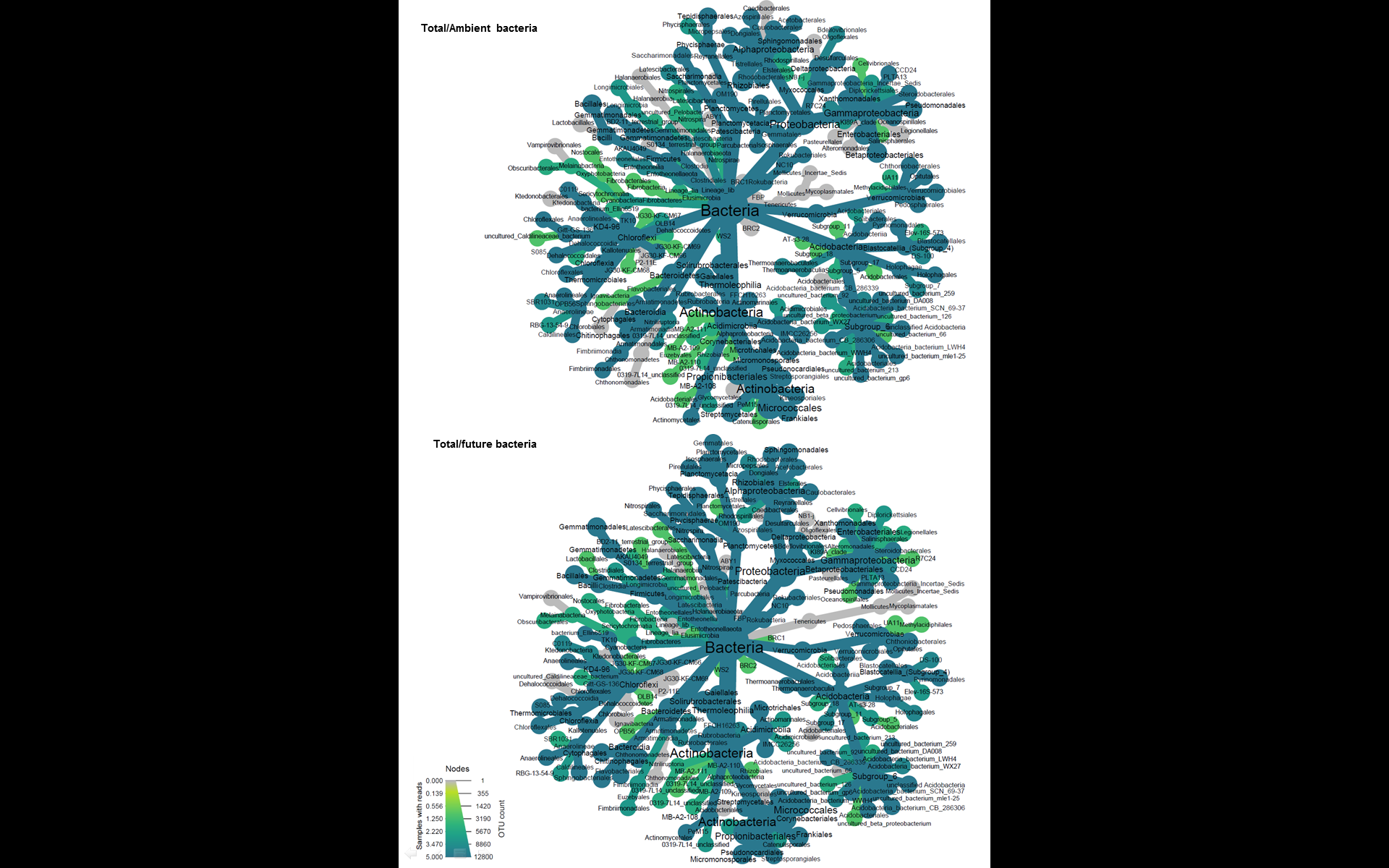


**Supplementary Figure S9.** Community composition of total bacteria under ambient and future climate regimes represented by heat tree, taxa are shown to order level.


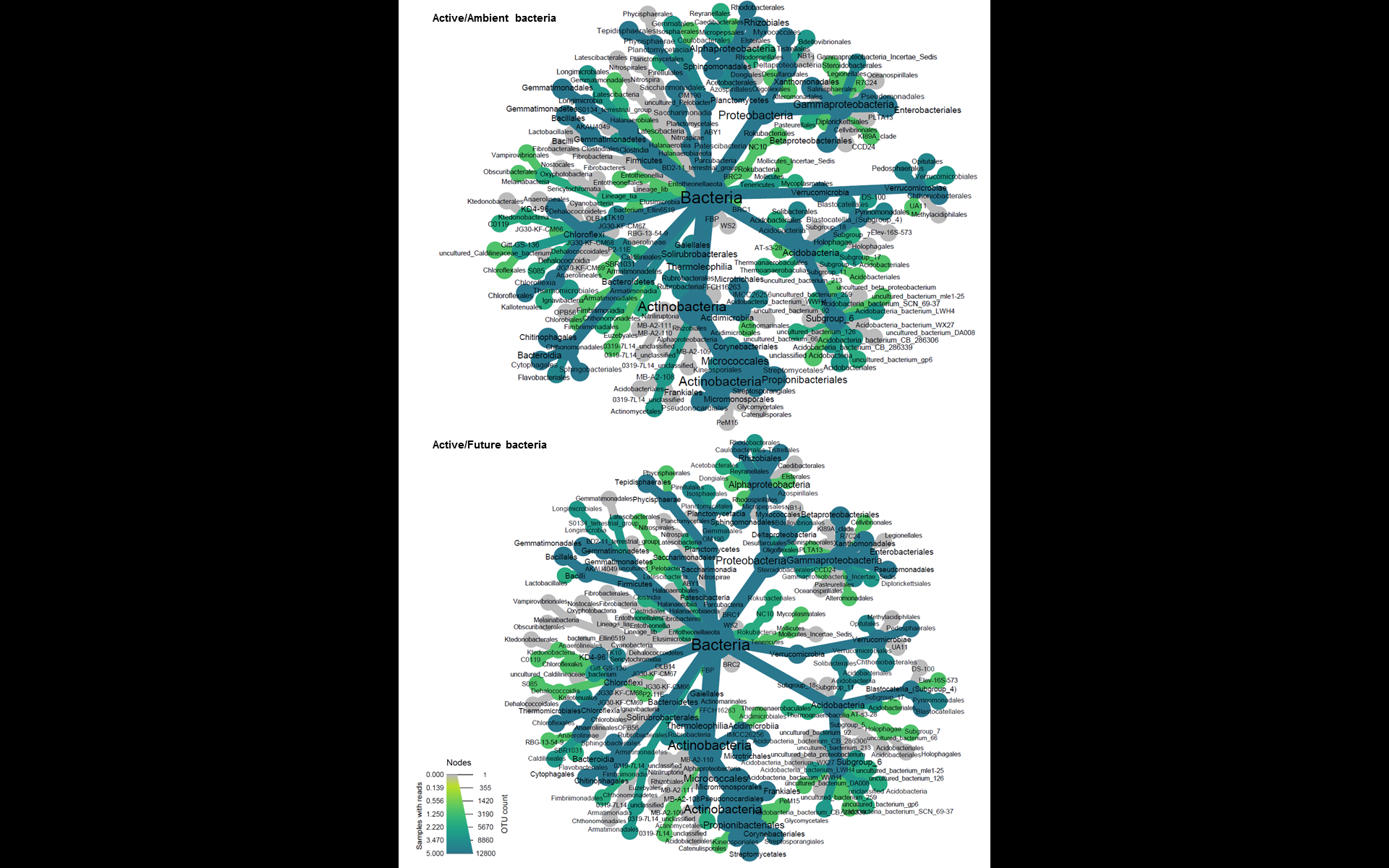


**Supplementary Figure S10.** Community composition of active bacteria under ambient and future climate regimes represented by heat tree, taxa are shown to order level.


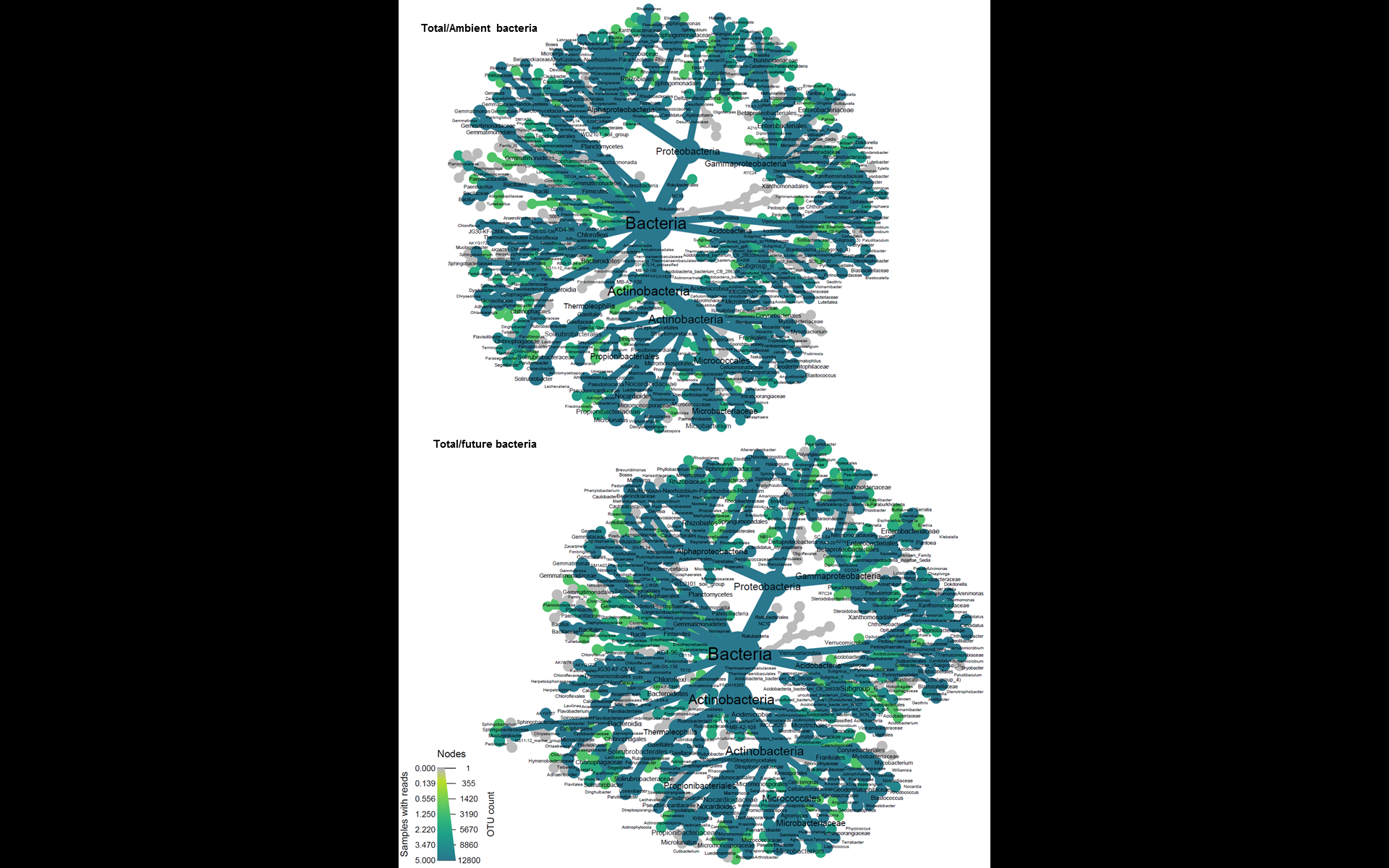


**Supplementary Figure S11.** Community composition of total bacteria under ambient and future climate regimes represented by heat tree, taxa are shown to genus level.


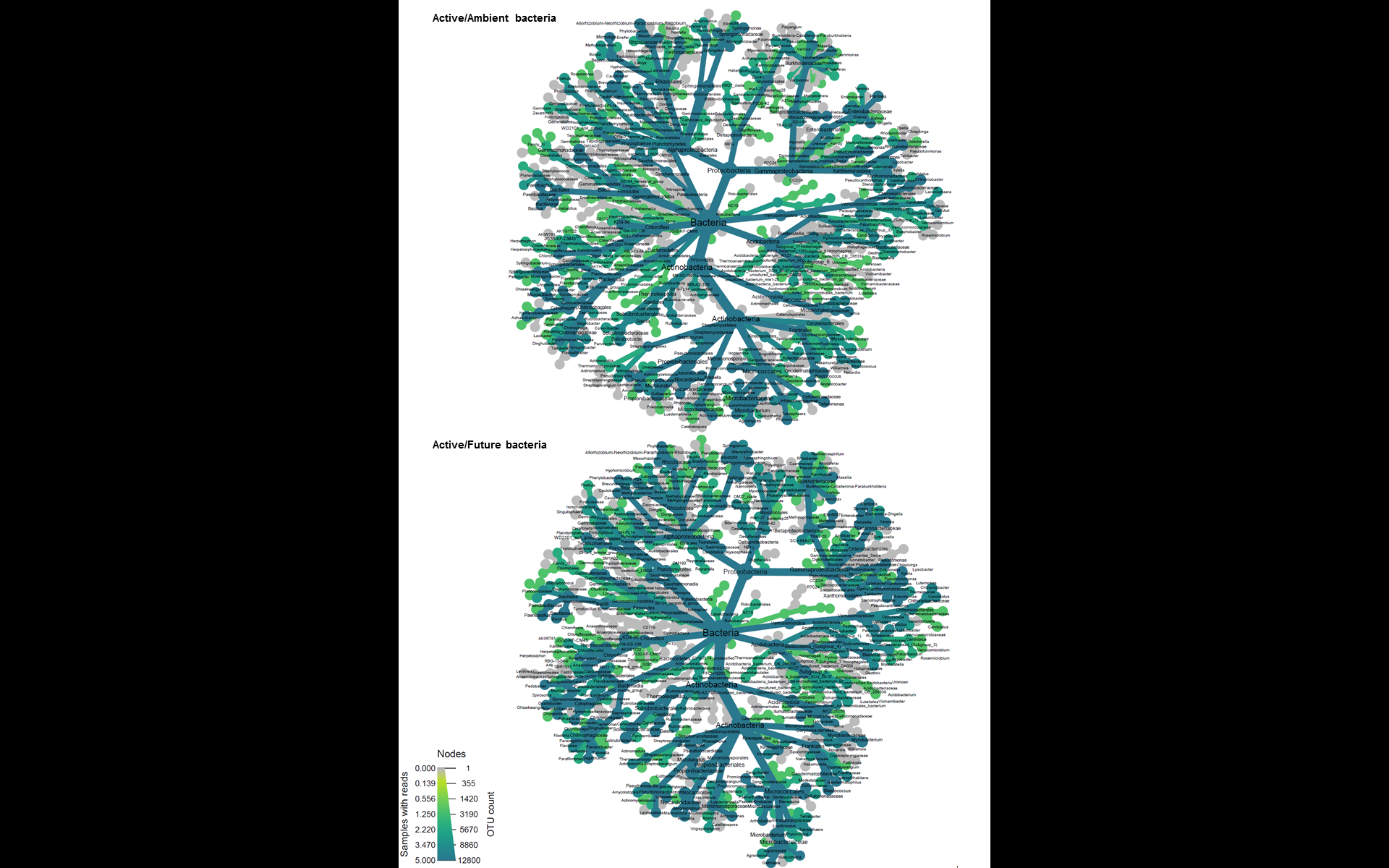


**Supplementary Figure S12.** Community composition of active bacteria under ambient and future climate regimes represented by heat tree, taxa are shown to genus level.


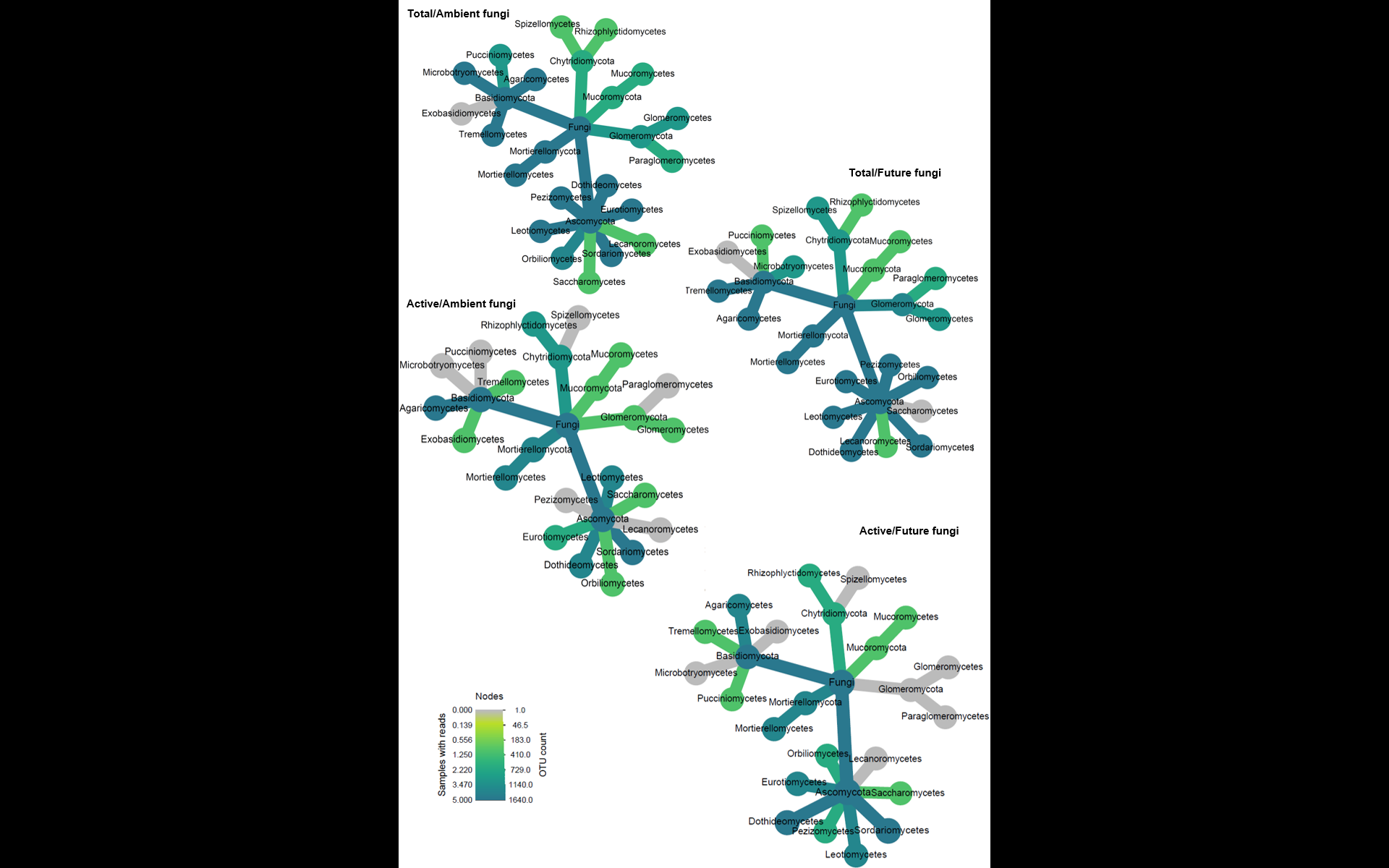


**Supplementary Figure S13.** Community composition of active and total fungi under ambient and future climate regimes represented by heat tree of overall fungal microbiome of each microbial fraction, taxa are shown to class level.


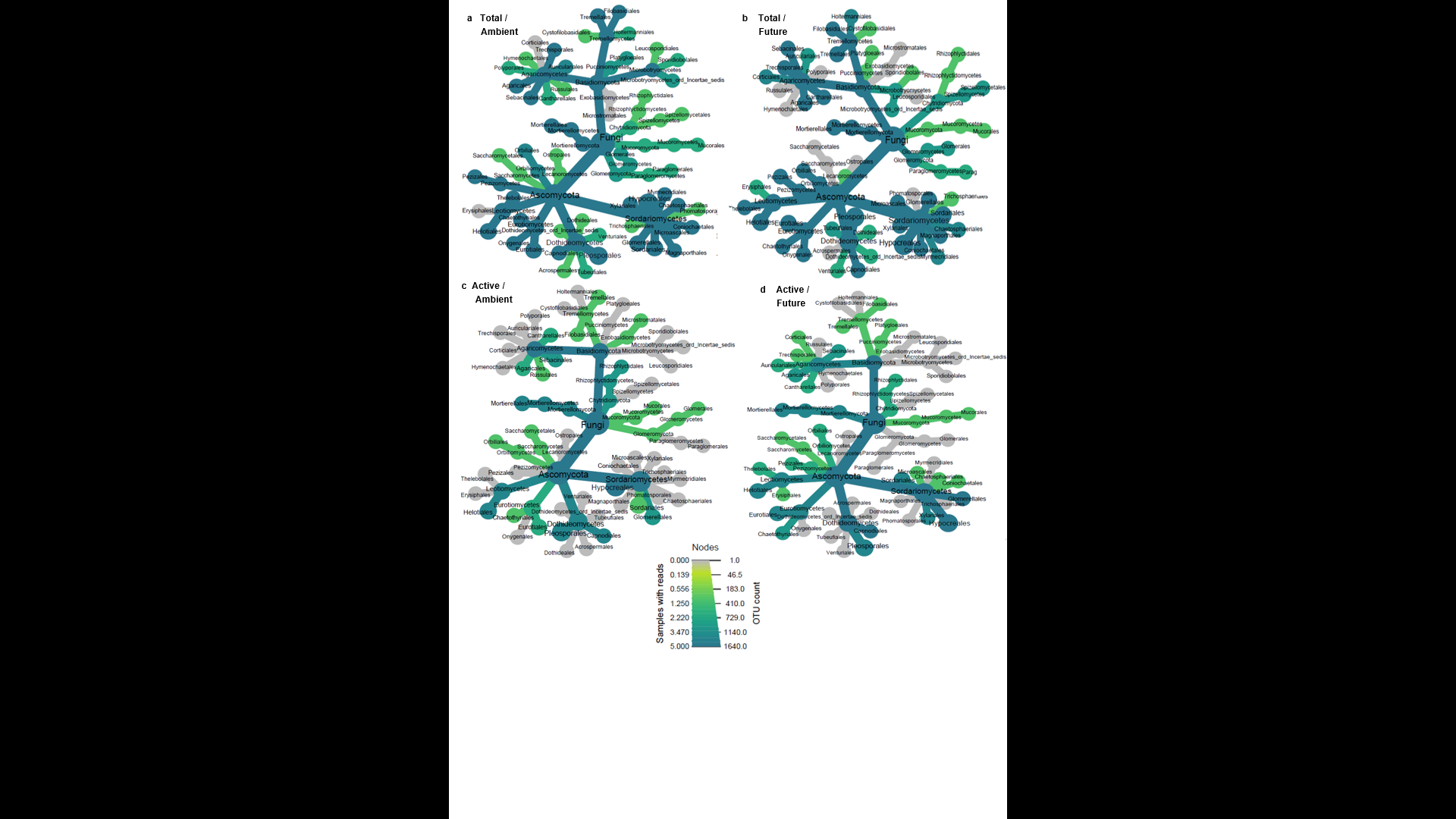


**Supplementary Figure S14.** Community composition of active and total fungi under ambient and future climate regimes represented by heat tree of overall fungal microbiome of each microbial fraction, taxa are shown to order level.

**
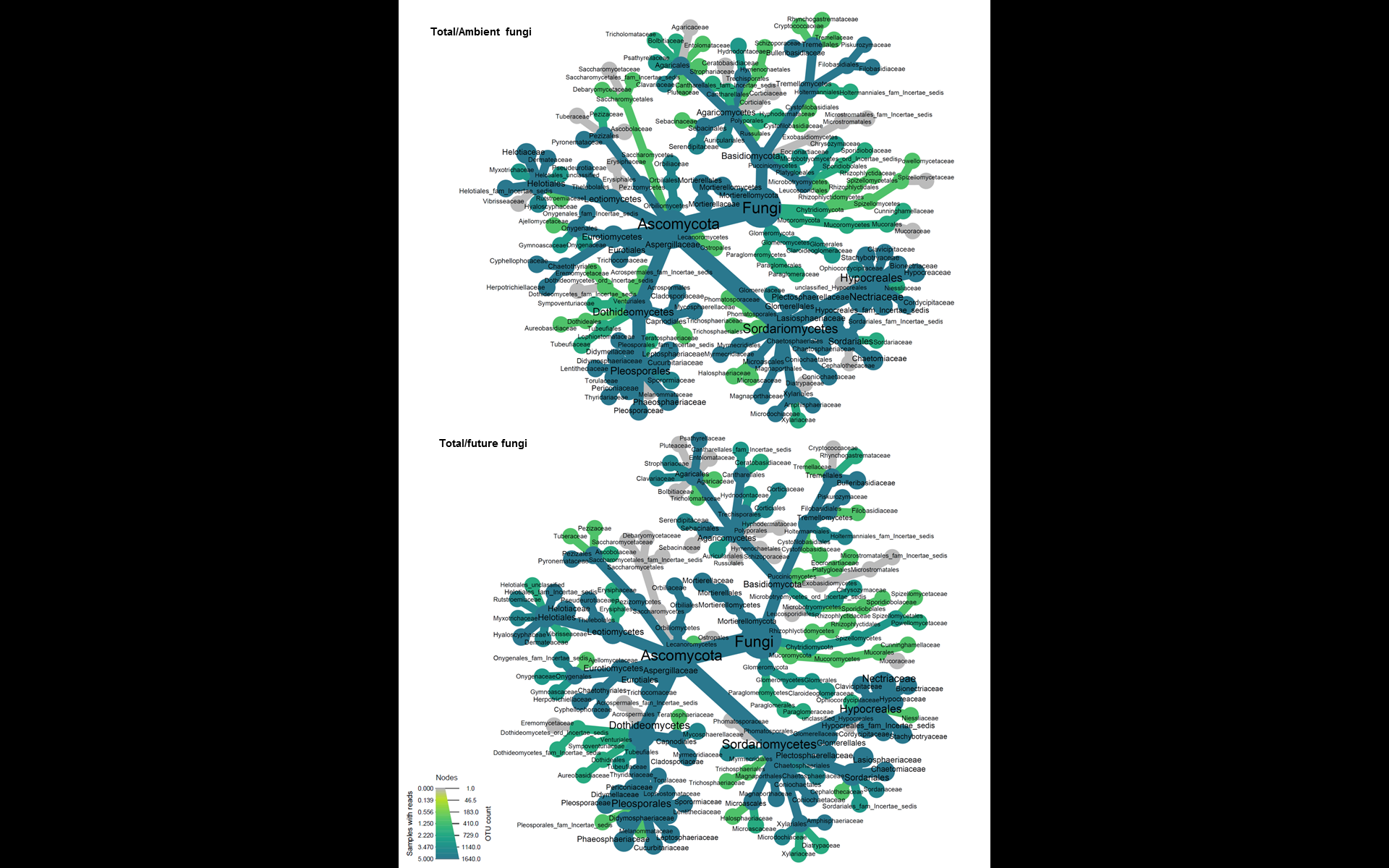
**

**Supplementary Figure S15.** Community composition of total fungi under ambient and future climate regimes represented by heat tree, taxa are shown to family level.


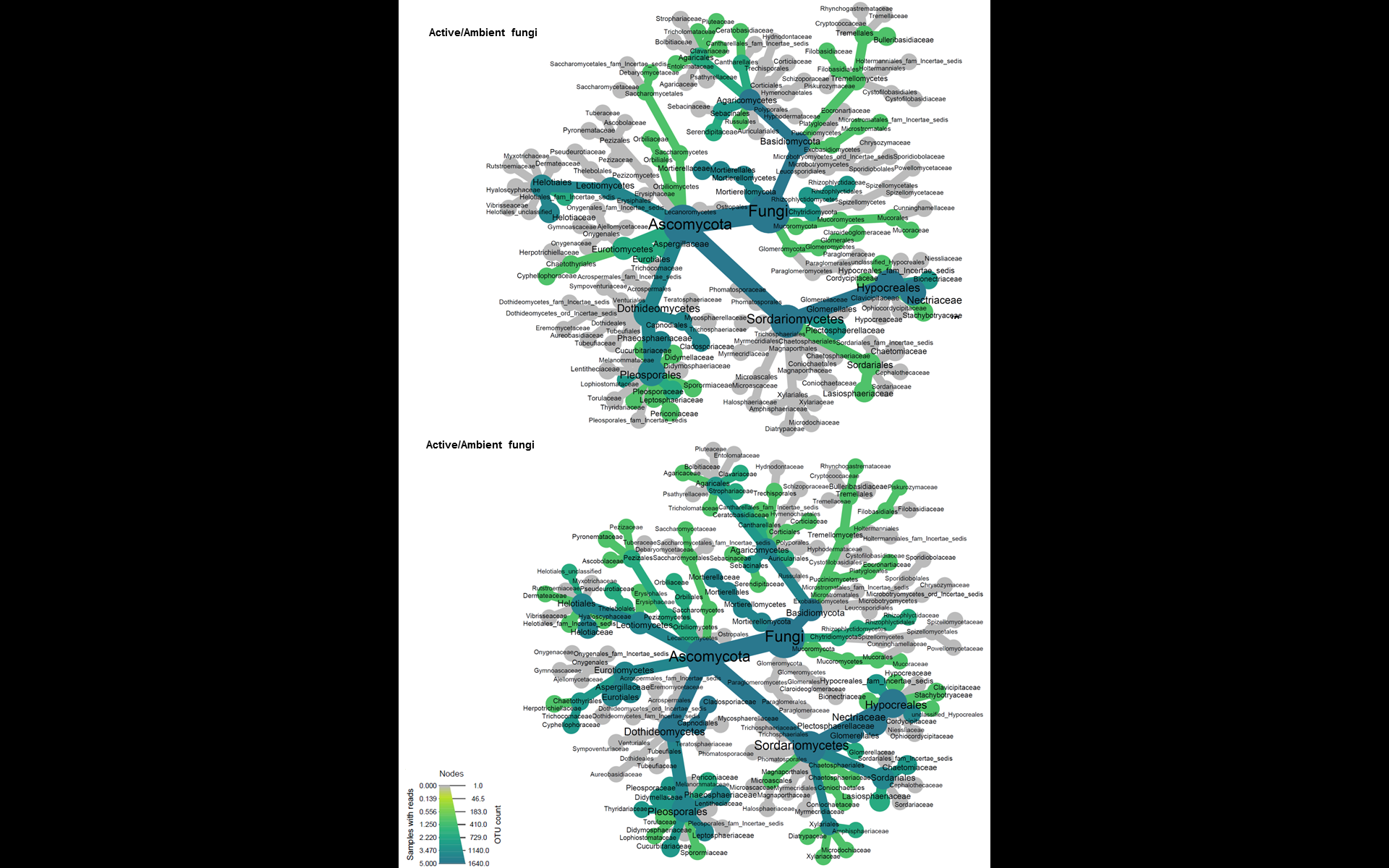


**Supplementary Figure S16.** Community composition of active fungi under ambient and future climate regimes represented by heat tree, taxa are shown to family level.


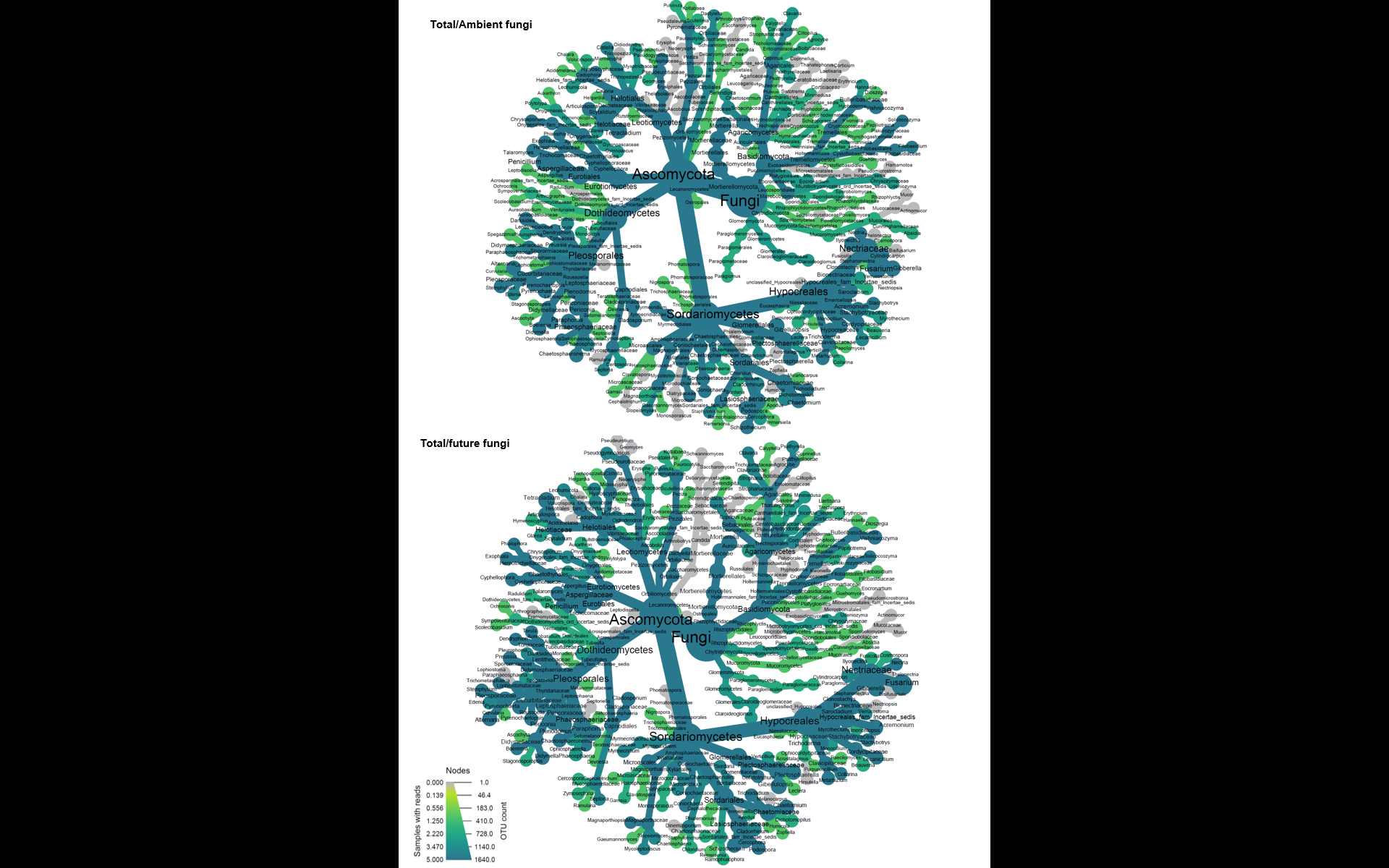


**Supplementary Figure S17.** Community composition of total fungi under ambient and future climate regimes represented by heat tree, taxa are shown to genus level.


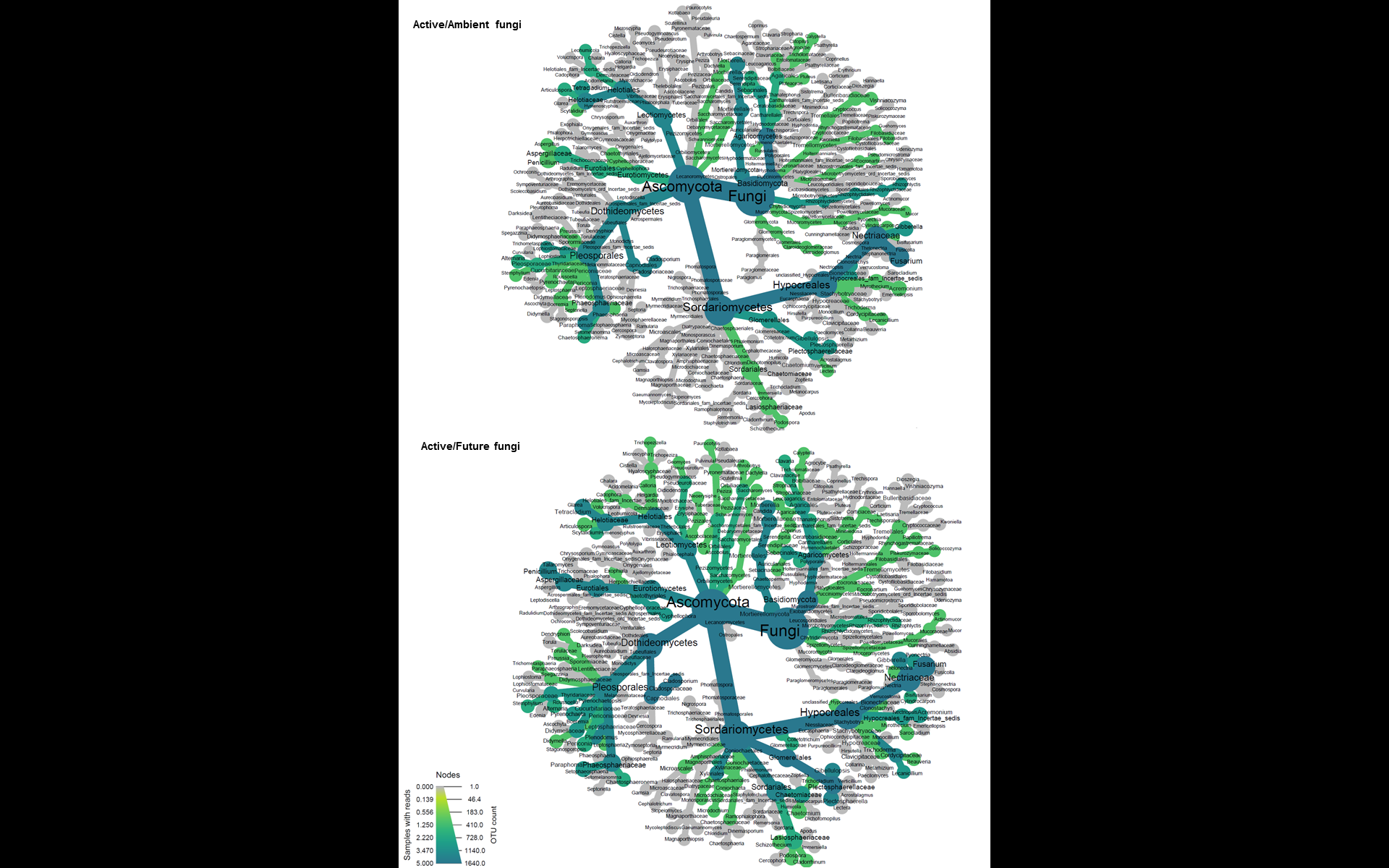


**Supplementary Figure S18.** Community composition of active fungi under ambient and future climate regimes represented by heat tree, taxa are shown to genus level.


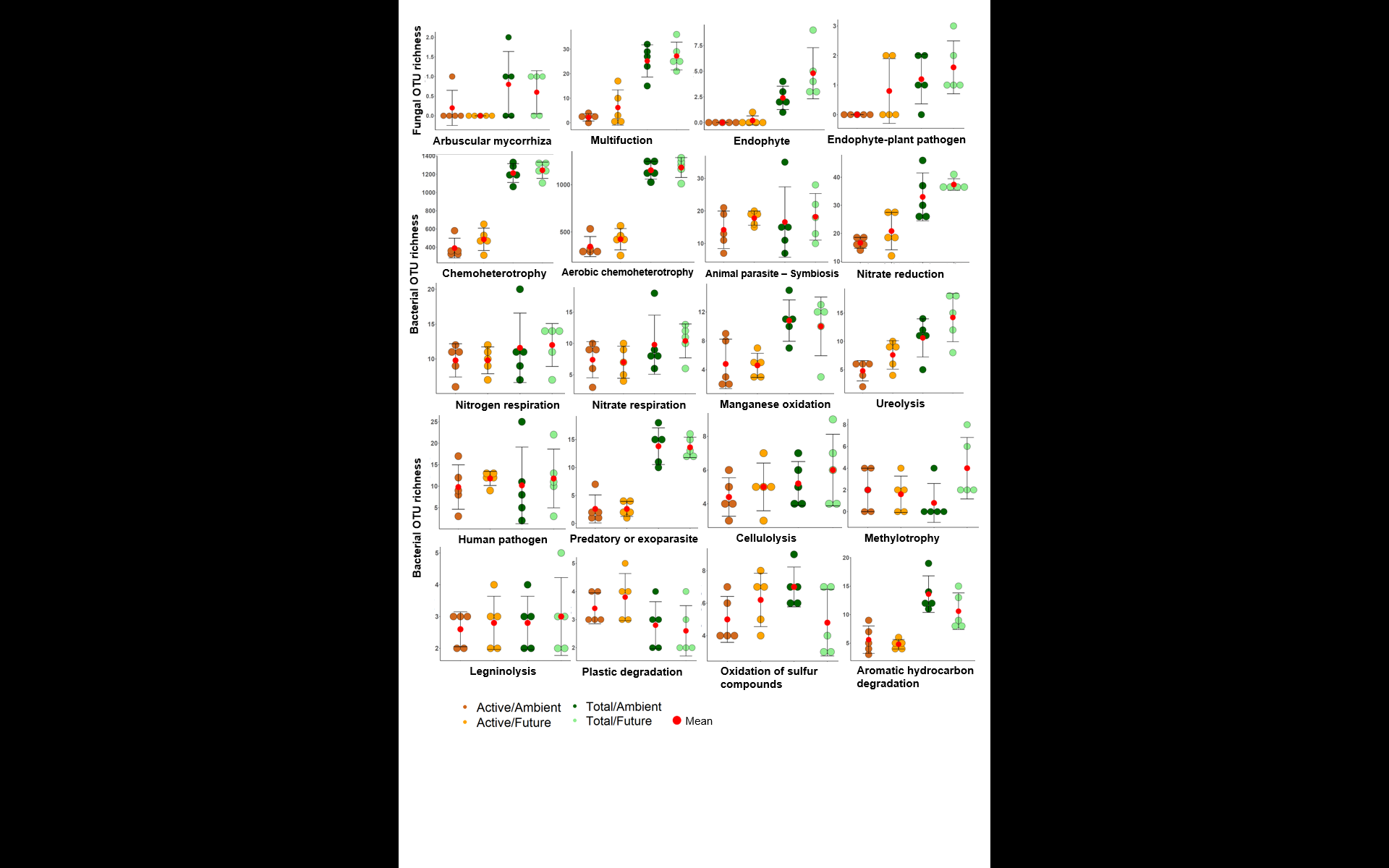


**Supplementary Figure S19.** OTU richness of predicted bacterial metabolic functions (FAPROTAX-based) and fungal guilds (FUNGuild-based) detected in active and total communities under ambient and future climate regimes.


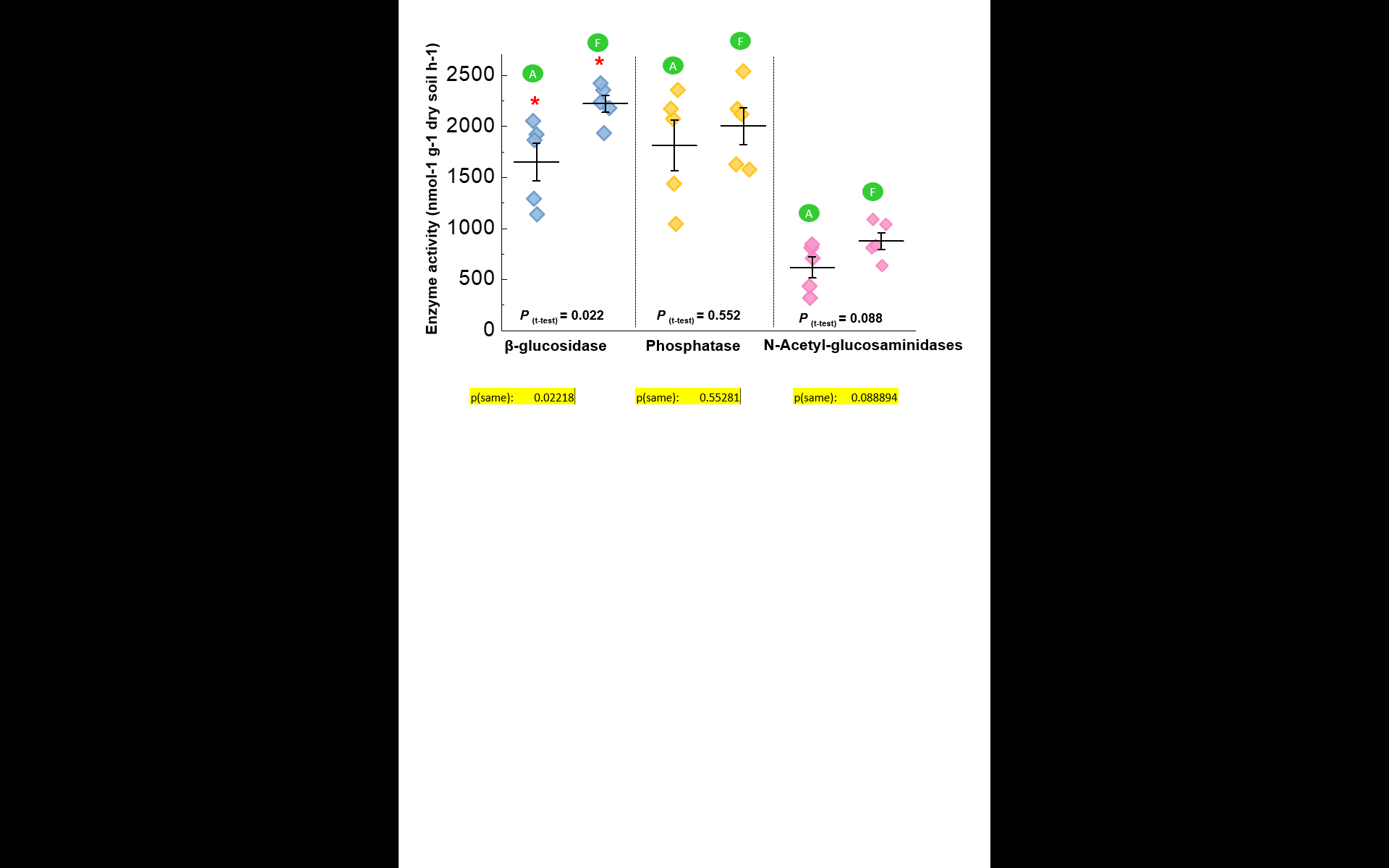


**Supplementary Figure** **S20.** Comparison between the activities of three extracellular enzymes under ambient and future climate regimes. A=ambient climate regime. F=future climate treatment.*significant influence of climate on enzyme activity.
